# Immunological memory to hyperphosphorylated tau in asymptomatic individuals

**DOI:** 10.1007/s00401-017-1705-y

**Published:** 2017-03-24

**Authors:** Gabriel Pascual, Jehangir S. Wadia, Xueyong Zhu, Elissa Keogh, Başak Kükrer, Jeroen van Ameijde, Hanna Inganäs, Berdien Siregar, Gerrard Perdok, Otto Diefenbach, Tariq Nahar, Imke Sprengers, Martin H. Koldijk, Els C. Brinkman-van der Linden, Laura A. Peferoen, Heng Zhang, Wenli Yu, Xinyi Li, Michelle Wagner, Veronica Moreno, Julie Kim, Martha Costa, Kiana West, Zara Fulton, Lucy Chammas, Nancy Luckashenak, Lauren Fletcher, Trevin Holland, Carrie Arnold, R. Anthony Williamson, Jeroen J. Hoozemans, Adrian Apetri, Frederique Bard, Ian A. Wilson, Wouter Koudstaal, Jaap Goudsmit

**Affiliations:** 1 Janssen Prevention Center, Janssen Pharmaceutical Companies of Johnson & Johnson, 3210, Merryfield Row, San Diego, CA 92121 USA; 2 Department of Integrative Structural and Computational Biology, The Scripps Research Institute, La Jolla, CA 92037 USA; 3 Janssen Prevention Center, Janssen Pharmaceutical Companies of Johnson & Johnson, Archimedesweg 6, 2333 CN Leiden, The Netherlands; 4 Department of Pathology, Amsterdam Neuroscience, VU University Medical Center, De Boelelaan 1117, Amsterdam, 1081 HV The Netherlands; 5 Janssen Prevention Center, Janssen Pharmaceutical Companies of Johnson & Johnson, 2 Royal College Street, London, NW1 0NH UK; 6 Skaggs Institute for Chemical Biology, The Scripps Research Institute, La Jolla, CA 92037 USA; 7 Department of Neurology, Amsterdam Neuroscience, Academic Medical Center, Meidreefberg 9, Amsterdam, 1105 AZ The Netherlands; 8 Department of Epidemiology, Harvard T.H. Chan School of Public Health, 677 Huntington Avenue, Boston, MA 02115 USA; 9 Janssen R&D US, 3210 Merryfield Row, San Diego, CA 92121 USA; 10 Informa, Pharma Intelligence, 3655 Nobel Drive, San Diego, CA 92122 USA; 11 TTP Labtech Inc, One Kendall Square, Cambridge, MA 02139-1594 USA; 12 Cidara Therapeutics, 6310 Nancy Ridge Drive, San Diego, CA 92121 USA

**Keywords:** Alzheimer’s disease, Tau protein, Memory B cell, Monoclonal antibody

## Abstract

Several reports have described the presence of antibodies against Alzheimer’s disease-associated hyperphosphorylated forms of tau in serum of healthy individuals. To characterize the specificities that can be found, we interrogated peripheral IgG^+^ memory B cells from asymptomatic blood donors for reactivity to a panel of phosphorylated tau peptides using a single-cell screening assay. Antibody sequences were recovered, cloned, and expressed as full-length IgGs. In total, 52 somatically mutated tau-binding antibodies were identified, corresponding to 35 unique clonal families. Forty-one of these antibodies recognize epitopes in the proline-rich and C-terminal domains, and binding of 26 of these antibodies is strictly phosphorylation dependent. Thirteen antibodies showed inhibitory activity in a P301S lysate seeded in vitro tau aggregation assay. Two such antibodies, CBTAU-7.1 and CBTAU-22.1, which bind to the proline-rich and C-terminal regions of tau, respectively, were characterized in more detail. CBTAU-7.1 recognizes an epitope that is similar to that of murine anti-PHF antibody AT8, but has different phospho requirements. Both CBTAU-7.1 and CBTAU-22.1 detect pathological tau deposits in post-mortem brain tissue. CBTAU-7.1 reveals a similar IHC distribution pattern as AT8, immunostaining (pre)tangles, threads, and neuritic plaques. CBTAU-22.1 shows selective detection of neurofibrillary changes by IHC. Taken together, these results suggest the presence of an ongoing antigen-driven immune response against tau in healthy individuals. The wide range of specificities to tau suggests that the human immune repertoire may contain antibodies that can serve as biomarkers or be exploited for therapy.

**Electronic supplementary material:**

The online version of this article (doi:10.1007/s00401-017-1705-y) contains supplementary material, which is available to authorized users.

## Introduction

Alzheimer’s disease (AD) is a progressive neurodegenerative disorder, characterized by neuronal loss and cognitive decline, which is rapidly becoming one of the leading causes of disability and mortality in the elderly [25, 26, 32, 61]. Two major pathological findings in patients with AD are extracellular plaques formed by amyloid β (Aβ) peptide [50, 51], and intracellular neurofibrillary tangles (NFT) containing aggregated tau protein [23, 36, 62]. Misfolding and aggregation of tau are closely associated with the onset and progression of AD and other related neurodegenerative disorders, such as progressive supranuclear palsy (PSP), corticobasal degeneration (CBD) and frontotemporal dementia (FTD), collectively referred to as tauopathies [41].

Tau is a highly soluble cytoplasmic protein expressed predominantly within the central nervous system (CNS) as six major isoforms, ranging in size from 352 to 441 amino acids. Under normal conditions, tau interacts with tubulin and plays an important role in maintaining microtubule architecture, thereby facilitating axonal transport and cytoskeletal remodeling [15, 21]. The tau protein sequence contains many potential phosphorylation sites, several of which have been shown to be important for modulating association with tubulin [8, 49]. Under pathogenic conditions, however, increased phosphorylation (known as “hyperphosphorylation”) occurs at these sites, promoting microtubule dissociation, aggregation into paired helical filaments (PHFs) and subsequently insoluble neurofibrillary tangles (NFTs), and impairment of axonal transport and synaptic function, which collectively drive neuronal toxicity and cell death (reviewed in [35]). Analyses of NFTs isolated from human AD cortical tissue have identified several important phosphorylation sites closely associated with disease pathology [60].

Consistent with this observation, phospho-dependent murine anti-tau monoclonal antibodies (mAbs), including AT8 and AT100, which were obtained following immunization with PHFs of tau isolated from AD brain tissue, are uniquely able to differentially recognize disease-related tau deposits that occur in AD and other tauopathies [4]. Naturally occurring antibody responses against self-antigens Aβ and tau have been reported in both healthy (noncognitively impaired) individuals and patients with AD [16, 18]. Recently, Dodel et al. have shown that antibodies to Aβ that preferentially bind to early oligomeric forms can be used to improve cognition in animal models of AD [14, 43]. In another study, serum IgM and IgG antibodies to unphosphorylated and phosphorylated tau peptides were detected in a survey of AD individuals and age-matched controls [48], and antibodies to both types of tau have been found in intravenous immunoglobulin (IVIG) preparations [29, 54, 55]. Very little is understood regarding the significance of these responses, although the presence of circulating Aβ and tau antibodies suggests that an ongoing autoimmune process may accompany AD.

In this study, we set out to assess what specificities of anti-tau antibodies could be recovered from human peripheral immune memory cells from apparently healthy donors. The completeness with which endogenous antibody repertoires can be described is proportionally related to the number of cells that can be interrogated. To screen immunological memory responses to tau efficiently, we used a high-throughput, single-cell screening methodology that allows multiparametric selection and recovery of antibodies from enriched human memory B-cell populations. Using this highly sensitive method, which we call BSelex, we report the presence of circulating tau-specific, IgG^+^ memory B cells in a high proportion of apparently healthy adult donor samples.

## Materials and methods

### Human PBMC isolation

Whole blood from healthy male and female donors was received from the San Diego Blood Bank (ages 18–65 years) or The Scripps Research Institute (ages 27–66 years). The use of samples from human volunteers followed protocols approved by the San Diego Blood Bank Review Board or The Scripps Research Institute Institutional Review Board depending on the donor source. Informed consent was obtained from the donors prior to the blood donation. Peripheral blood mononuclear cells (PBMCs) were isolated on Ficoll-Paque Plus (GE Healthcare) and cryopreserved at 50 million cells per ml in 90% FBS and 10% DMSO.

### Peptide synthesis

In an effort to identify and recover naturally occurring human mAbs to tau, a panel of 122 overlapping, phosphorylated peptides spanning all putative phosphorylation sites across human tau isoform 2N4R (tau441) was designed and synthesized. The overlapping tau phosphopeptides ranged from 18 to 26 amino acids, containing either one or two phosphorylated residues to represent all possible combinations in regions with multiple putative phosphorylation sites. An amino-terminal biotin group was added during synthesis to facilitate capture by streptavidin in downstream experiments. An additional ten nonphosphorylated peptides spanning the length of tau441 were synthesized. Each peptide was synthesized by solid-phase chemistry with >95% purity (New England Peptide, Inc.).

### Generation of tau-peptide baits and single cell sorting of tau-specific memory B cells

Tau-peptide baits were prepared by mixing biotinylated peptides or biotin (negative control) with streptavidin-APC or streptavidin-PE (Thermo Fisher) at a 35:1 molar ratio. The mixture was incubated overnight at 4 °C with gentle mixing and passed over a BioSpin 30 column (Biorad) to remove free peptide or biotin. PBMCs from 3–4 donors were thawed quickly, transferred to tubes containing prewarmed RPMI complete (RPMI, 10% heat-inactivated FBS and 1% pen/strep), washed, and incubated separately at 37 °C for 16 h. The cells were collected and the B cells enriched by positive selection with CD22^+^ magnetic beads (Miltenyi Biotec). Cells were resuspended in Tris buffered saline, pH 7.4, containing 2 mM EDTA and 0.25% bovine serum albumin, Fraction V (TBS buffer). An aliquot was removed for cytometer controls, and the extracellular markers IgG-FITC, CD19-PerCPCy5.5, and CD27-PECy7 (all from BD Biosciences) were added to the remaining cells. Ten million cells were removed for the negative control, and the biotin–streptavidin conjugates were added. The remaining cells were subsequently incubated with a pool of dual labeled peptide tetramers. The background and experimental tubes were incubated for 60 min at 4 °C with gentle mixing, washed and resuspended at 20 million cells per ml in TBS buffer. Prior to sorting, DAPI (Thermo Fisher) was added as a live/dead marker, and the cells were sorted on a Beckman Coulter MoFlo XDP. The negative control was used to determine nonspecific binding, to set the gates, and to determine the signal-to-noise ratio. Doublets and dead cells were excluded, and the CD19^+^, IgG^+^, CD27^hi^, and antigen double-positive cells were collected by single cell sorting. Cells were collected directly into PCR plates containing RT-PCR reaction buffer and RNaseOUT (Thermo Fisher). Plates were sealed with LMT-Aluma II covers (Phenix Research Products), centrifuged at 1000 rpm for 1 min, and immediately frozen at −80 °C.

### Recovery of heavy and light chain antibody genes from tau-specific memory B cells

Heavy and light chain (HC/LC) antibody variable regions were recovered using a two-step PCR approach from single-sorted memory B cells. Variable chain fragments were subsequently cloned and expressed as human IgG1. cDNA was first synthesized according to manufacturer’s instructions (Thermo Fisher, Superscript III First Strand Synthesis kit), and 2.5 µl of cDNA sample was immediately used for step I HC/LC PCR reactions. A pool of forward primers specifically designed to the HC/LC antibody leader sequences and a single reverse primer specific to either Cγ, Cκ, or Cλ was used in the step I reaction. Step II PCR reactions were then prepared using 2.5 µl of the step I PCR product as template using a pool of forward primers specific to the HC and LC (κ and λ) framework 1 region and a pool of reverse primers specific to the junction region of each antibody chain. Step II PCR fragments, ranging from 380 to 400 kb in size, were resolved and purified. Finally, the Step II fragments were linked via overlap extension PCR using a 1.8 kb linker product. Overlapped HC/LC products were subsequently digested (XbaI and XhoI, New England Biolabs) and cloned into a dual-CMV–based human IgG1 mammalian expression vector (generated in house).

### Recombinant IgG expression

Cloned anti-tau human mAbs were transiently transfected in human embryonic kidney 293-derived Expi293 cells (Thermo Fisher) and 72 h post transfection, cell media were harvested and centrifuged for 7 min at 1200 RPM. IgG was purified from the culture medium by standard Protein A affinity chromatography methods. IgGs were eluted from the protein A affinity column in 100 mM sodium citrate buffer, pH 3.5 which was immediately exchanged for PBS. Antibodies were quantitated using Protein A sensor tips on the Octet Red384 (ForteBio). Each antibody was quality controlled by SDS-PAGE and size-exclusion chromatography, and was further confirmed for reactivity to cognate tau peptide by ELISA. For immunohistochemistry, mouse chimeric versions of CBTAU-7.1 and CBTAU-22.1 were generated by replacing the human Fc with a murine Fc.

### Aggregation assay

All animal work was done in accordance with local Institutional Animal Care and Use Committee guidelines and was reviewed and approved by the on-site Janssen R&D Institutional Animal Care and Use Committee. T43 transgenic mice express human 0N/4R tau containing the P301S mutation from the Thy1 promoter [3]. At 5–6 months of age, aggregated, hyperphosphorylated tau accumulates in the brain and spinal cord of these mice. Spinal cord tissues were harvested from 6-month-old transgenic mice and snap frozen. Extracts were prepared by sonication of tissue in TBS with protease inhibitors (Roche Applied Science), and total protein was quantified using Pierce™ BCA Protein Assay Kit (Thermo Fisher). The biosensor cell line containing the cyan fluorescent protein (CFP)- and yellow fluorescent protein (YFP)-tagged tau repeat domain (RD) reporters was obtained from Marc Diamond [28]. For the assays, cells were plated at 10,000 cells/well in 96 well plates in 130 µL DMEM (with 10% FBS, glutamax, and pen/strep) and allowed to attach for 24 h. Spinal cord extract from P301S tau transgenic mice was preincubated with various concentrations of antibodies, or buffer alone, for 2 h at 37 °C before addition to the cell culture media. The tau extract/antibody mixtures were incubated on the cells for 72 h. For harvest and analysis, the cells were trypsin treated, washed, fixed in 2% paraformaldehyde for 10 min, and then resuspended in 1% FBS, 1 mM EDTA in HBSS for flow cytometry analysis on a MACSQuant 10. After gating on the cell population using FSC-SSC, and eliminating doublet cells, cells that were double-positive for CFP and YFP were selected for further analysis. From those cells, the percent of cells with a fluorescence resonance energy transfer (FRET) signal was measured. The FRET responses for each antibody, normalized against tau extract only, were collected from the results of several assays; results are shown for the highest concentration tested in two or more replicate assays. To facilitate the interpretation of the data, the results were expressed as the reduction in FRET response (i.e., 100—normalized FRET). Variation between assays was estimated (SD = 3.96%) and used to set a cutoff (at 0% + 2 × SD = 7.92%). Ultimately, results were expressed as the mean reduction in FRET response (across all assays). For antibodies CBTAU-7.1, CBTAU-22.1 and AT8, a dose–response relationship was determined by serial dilution.

### Reactivity of anti-tau human mAbs to tau peptides by ELISA

Biotinylated tau peptides were captured on streptavidin-coated plates (Thermo Fisher) at 1 µg/ml in TBS and incubated for 2 h. 96-well half-area ELISA plates (Costar) were coated overnight at 4 °C with goat anti-human Fab (2 µg/ml, Jackson ImmunoResearch) to measure total IgG. Bovine actin and an irrelevant (non-cognate) peptide chosen from the panel of 122 tau peptides were coated at 1 µg/ml in TBS and used to confirm specificity of the purified mAbs. ELISA plates were washed four times with TBS/0.05% Tween 20 (TBS-T) and blocked with 2.5% BSA in TBS for 2 h. Purified IgGs were diluted to 5 µg/ml in TBS/0.25% BSA and titrated (fivefold serial dilutions) against peptides. Diluted samples were added to the plates and incubated for 2 h at room temperature. A human chimeric version of the PHF-tau-specific mouse antibody AT8 was generated by swapping out the mouse heavy and light chain constant regions for human IgG1 sequence. This AT8 chimeric antibody, which reacts to a phosphopeptide (Ser202/Thr205) spanning region 194–212, was used as a positive control at 1 µg/ml in ELISA assays when this phosphopeptide was tested. The plates were washed five times with TBS-T and secondary antibodies diluted in TBS/0.25% BSA were added and incubated at room temp for 1 h. Goat anti-human IgG F(ab’)_2_ (Jackson ImmunoResearch) was used at 1:2000 dilution and goat anti-mouse HRP (Jackson ImmunoResearch) was used at 1:4000 dilution. Following incubation, plates were washed four times in TBS-T and developed with Sure Blue Reserve TMB Microwell Peroxidase Substrate (KPL) for approximately 2 min. The reaction was stopped by the addition of TMB Stop Solution and the absorbance at 450 nm was measured using an ELISA plate reader.

### Western blot analysis

Reactivity to recombinant tau and PHF was tested by Western blot analysis. Immunopurified PHF was provided by Steven Paul at Weill Cornell Medical College and prepared as previously described [22, 33]. Approximately 0.3 µg of PHF and 0.2 µg of recombinant tau (Sigma) were resolved on SDS-PAGE (4–12% Bis–Tris Novex NuPAGE gel; Invitrogen) and subsequently transferred onto a nitrocellulose membrane. The membrane was blocked overnight in 1X TBS-T with 5% BSA (blocking buffer). Human chimeric anti-tau antibodies AT8 and htau10 were used as control antibodies at 25 µg/ml in 2.5% BSA in TBS-T and incubated for 2 h at room temperature. The human chimeric version of htau10 was generated, as described above for chimeric AT8, by swapping out the mouse heavy and light chain constant regions for human IgG1 sequence. The membranes were then washed three times for 5 min each in TBS-T. Peroxidase AffiniPure goat anti-human IgG (Fc-γ fragment specific; Jackson ImmunoResearch) was used as secondary antibody at a 1:4000 dilution in 2.5% BSA in TBS-T and incubated for 1 h at room temperature. The membrane was washed three times for 5 min and developed using the Supersignal West Pico kit (Pierce). Images were obtained on the ImageQuant LAS-4000 (GE Healthcare).

### Immunoprecipitation–mass spectrometry (IP-MS)

To avoid contamination of the final eluate with IgG, a chemical crosslinker (BS3, Thermo Fisher) was employed to covalently link the CBTAU mAbs to magnetic Protein A beads (Dynabeads, ThermoFischer Sci.). 25 μg of CBTAU was used per 100 μL bead suspension, with a crosslinker concentration of 0.5 mM. The prepared beads were added to 10 μL P301S transgenic mice brainstem homogenate (total protein concentration 6.8 mg/mL, determined as described above) and incubated overnight at 4 °C after which flow-through was collected. The beads were extensively washed to remove any nonspecific interactors and final elution was performed with LDS (Novex Nupage; Thermo Fisher) at 70 °C for 10 min. It should be noted that the affinities of phosphorylation-dependent antibodies 7.1 and 22.1 are highly dependent on ionic strength and, for these IgGs, the protocol was carried out with buffers having a tenth of the ionic strength of PBS. The flow-through, wash, and elution fractions were separated by SDS-PAGE (4–12% Bis–Tris Novex NuPAGE gel; Thermo Fisher) and analyzed by Western blot on PVDF membranes (iBlot; Invitrogen) using total-Tau HT7 (Thermo Fischer) at a 1:1000 dilution as primary antibody and 1:25,000 goat anti-mouse HRP conjugate (Invitrogen) as secondary antibody. ECL staining (GE Healthcare) was performed according to the manufacturer’s protocol. A separate elution was also performed with 1% aqueous formic acid. Removal of acidic solution by refrigerated CentriVap benchtop vacuum concentrator (Labconco, US) has been followed by in-solution trypsin digestion of the eluted samples. The resulting tryptic peptides were separated by reverse-phase chromatography using Waters nano-ACQUITY UPLC pumps in combination with a column switching system. Trapping of the peptides was performed using a Waters BEH C18 column (1.7 mm particles, 2.1 mm × 5 mm). Subsequently, peptides were separated by a Waters UPLC BEH C18 analytical column (1.7 mm particles, 1 mm × 100 mm) using gradient elution. Mass and fragmentation data of the separated peptides were recorded with a Waters Xevo-G2S QTof Mass Spectrometer operating in the MS^e^ mode. Recorded data has been analyzed by the Waters BiopharmaLynx software (version 1.3.3). In addition, manual inspection of the spectra was performed using the MassLynx Software (version 4.1) to confirm the identity of peptides.

### Peptide mapping by ELISA

Streptavidin-coated 96-well plates (Thermo Fisher) were washed four times with TBS-T, followed by incubation overnight at 4 °C with 400 nM of the panel of tau-specific biotinylated peptides in Table S3. Plates were washed four times the following day with TBS-T and subsequently blocked for 1 h with 2.5% BSA in TBS. Human anti-tau antibodies were added at 2 µg/ml in TBS/0.25% BSA for 1 h at room temperature. Plates were washed four times with TBS-T, followed by incubation with goat anti-human IgG F(ab’)_2_ (Jackson ImmunoResearch) at 1:2000 dilution in TBS/0.25% BSA for 1 h at room temperature. Plates were washed six times in TBS-T and developed with Sure Blue Reserve TMB Microwell Peroxidase Substrate (KPL) for approximately 90 s. The reaction was stopped by addition of TMB Stop Solution, and absorbance at 450 nm was measured using an ELISA plate reader. Nonspecific binding of the human anti-tau mAbs was determined by measuring binding to non-peptide coated wells and subtracted for each antibody.

### Qualitative association and dissociation measurements by Octet biolayer interferometry

The relative binding of CBTAU-7.1 and CBTAU-22.1 mAbs to tau peptides corresponding to their assumed epitope was assessed by biolayer interferometry (Octet Red 384) measurements (ForteBio) [11]. Biotinylated tau peptides were immobilized on Streptavidin (SA) Dip and Read biosensors for kinetics (ForteBio). Real-time binding curves were measured by applying the sensor in buffer containing 100 nM CBTAU mAbs. The immobilization of peptides to sensors and the association were followed in different ionic strength buffers containing 10% kinetic buffer. To induce dissociation, the biosensor containing the mAb-tau-peptide complex was immersed in kinetic buffer (ForteBio) without antibody. The relative association and dissociation kinetic curves were compared to qualitatively assess the efficiency of CBTAU binding to peptides with different phosphorylation patterns under different conditions.

### Affinity measurements by isothermal titration calorimetry (ITC)

The affinities of AT8, CBTAU-7.1, and CBTAU-22.1 for their corresponding tau peptides were determined in solution on a MicroCal Auto iTC200 system (Malvern). Peptides at concentrations of ~30 µM (AT8), ~40 µM (CBTAU-7.1), and ~40 µM (CBTAU-22.1) were titrated in 20 steps of 2 µl per step in identical buffers containing 140 µM AT8, 200 µM CBTAU-7.1 and 200 µM CBTAU-22.1, respectively. The thermodynamic parameters and the equilibrium dissociation constants, Kd, were determined upon fitting the ITC data to a model assuming a single set of binding sites corresponding to a mAb:tau peptide = 1:2 binding model.

### Expression of recombinant CBTAU-7.1 and CBTAU-22.1 Fabs for crystallization

To express Fab fragments for crystallization, the hinge, C_H_2, and C_H_3 sequences of the cloned CBTAU-7.1 and CBTAU-22.1 IgGs in the dual-CMV-based human IgG1 mammalian expression vector were removed and a His_6_ tag added to the C-terminus of C_H_1. The Fab fragments were produced by transient transfection of FreeStyle 293-F cells and purified using an Ni–NTA column followed by size-exclusion chromatography using a Superdex 200 column (GE Healthcare).

### Crystallization, data collection, and structure determination

Crystallization of CBTAU-7.1 and CBTAU-22.1 Fabs was performed using the high-throughput automated CrystalMation robot (Rigaku) at The Scripps Research Institute (TSRI). Following optimization, CBTAU-7.1 crystals were obtained using the vapor diffusion sitting drop method at 20 °C by mixing 0.1 µl of the concentrated protein (8 mg/ml) in 20 mM Tris, pH 8.0, 150 mM NaCl, 0.02% NaN_3_ with 0.1 µl of a reservoir solution containing 20% (w/v) polyethylene glycol (PEG) 3350, 0.2 M ammonium dihydrogen phosphate. Before data collection, the CBTAU-7.1 crystals were soaked for a few seconds in the reservoir solution supplemented with 15% (v/v) glycerol and then flash-frozen in liquid nitrogen. CBTAU-22.1 crystals were obtained using the same method at 20 °C by mixing 0.1 µl of the concentrated protein (12 mg/ml) in 20 mM Tris, pH 8.0, 150 mM NaCl, 0.02% NaN_3_ in the presence of 0.9 mM of tau-peptide ^412^SSTG(pS)IDMVD(pS)PQLATLA^429^ (pS, phosphorylated serine) with 0.1 µl of a reservoir solution containing 0.1 M citrate buffer, pH 4.0, 2.4 M (NH_4_)_2_SO_4_. Before data collection, the CBTAU-22.1 crystals were soaked for a few seconds in the reservoir solution supplemented with 25% (v/v) glycerol and then flash-frozen in liquid nitrogen. The X-ray structures of the CBTAU-7.1 and CBTAU-22.1 Fabs were determined by molecular replacement (MR) using the program Phaser [42]. The initial models for MR of CBTAU-7.1 Fab were the Fab N12-I2 light chain (PDB code 3QEG) and Z13E1 Fab heavy chain (PDB code 3FN0). The initial models for MR of CBTAU-22.1 Fab were the Fab MSL-109 light chain (PDB code 4LRI) and putative VRC01 germline precursor Fab heavy chain (PDB code 4JPK). One Fab was found in the asymmetric unit in both structures. Initial rigid body refinement was performed using program Phenix [1]. Further model rebuilding was performed using the graphics program Coot [17] and refined with Phenix. As no electron density for bound peptide was present in the CBTAU-22.1 electron density, the peptide ligand was presumably outcompeted by the high molarity of sulfate ions from the 2.4 M ammonium sulfate in the crystallization condition. Final refinement statistics are summarized in Table S4.

### Post-mortem human brain tissue

Brain samples were obtained from The Netherlands Brain Bank (NBB), Netherlands Institute for Neuroscience, Amsterdam (open access. www.brainbank.nl). All materials were collected from donors for or from whom written informed consent for a brain autopsy and the use of the material and clinical information for research purposes had been obtained by the NBB. Dementia status at death was determined on the basis of clinical information available during the last year of life and neuropathological diagnosis using histochemical stains (haematoxylin and eosin, Bodian and/or Gallyas silver stains [59], methenamine silver stain) and immunohistochemistry for Aβ, p-tau (AT8), α-synuclein, TDP43, and P62. Analysis of formalin-fixed and paraffin-embedded tissue from different parts of the brain was performed, including the frontal cortex (F2), temporal pole cortex, parietal cortex (superior and inferior lobule), occipital pole cortex, amygdala, and the hippocampus, essentially CA1 and entorhinal area of the parahippocampal gyrus. Staging of pathology was evaluated according to a modified assessment of Braak and Alafuzoff and the Aβ staging of Thal [2, 9, 57]. For cases with and without clinical neurological disease, diagnosis was processed identically. Brain tissue samples were either formalin-fixed and paraffin embedded or snap frozen (liquid N2). For this study, brain tissue was obtained from the hippocampus (middle part at the level of the lateral geniculate body), caudate nucleus, the frontal cortex (F2, premotor dorsal, caudal), and the mid-temporal cortex.

### Immunohistochemistry

Slide-mounted immunohistochemistry: Sections (5 μm thick) from formalin-fixed paraffin-embedded tissue were mounted on Superfrost Plus tissue slides (Menzel-Gläser, Germany) and dried overnight at 37 °C. Sections were deparaffinized and subsequently immersed in 0.3% H_2_O_2_ in phosphate-buffered saline (PBS) for 30 min to quench endogenous peroxidase activity. Sections were treated in sodium citrate buffer (10 mM sodium citrate, pH 6.0) heated by autoclave (20 min at 130 °C) for antigen retrieval. Between the subsequent incubation steps, sections were washed extensively with PBS. Primary antibodies were diluted in antibody diluent (Immunologic) and incubated overnight at 4 °C. Secondary EnVison^TM^ HRP goat anti-rabbit/mouse antibody (EV-GαM^HRP^, DAKO) was incubated for 30 min at RT. 3,3-Diaminobenzidine (DAB; DAKO) was used as chromogen. Sections were counterstained with haematoxylin to visualize the nuclei of the cells, dehydrated and mounted using Quick-D mounting medium (BDH Laboratories Supplies, Poole, England). To remove phosphate groups from phosphorylated proteins prior to IHC detection, sections were treated with alkaline phosphatase solution before incubation with primary antibodies. Before incubation, sections were washed with Tris–HCl, pH 8.0 and incubated in freshly prepared alkaline phosphatase solution (100 mM Tris–HCl, pH 8.0 supplemented with 130 units/mL calf intestinal alkaline phosphatase (New England Biolabs), 1 mM phenylmethylsulfonyl fluoride (PMSF) (Sigma), 10 µg/mL pepstatin (Sigma), 10 µg/mL leupeptin (Sigma)) for 2.5 h at 37 °C. Sections were rinsed twice with 100 mM Tris–HCl, pH 8.0 for 3 min at RT prior to detection with primary antibodies. Double labeling of paraffin-embedded tissue sections was carried out as follows. Deparaffinized sections were subjected to wet autoclave antigen retrieval in citrate buffer and incubated overnight with chimeric CBTAU-7.1 (2.5 μg/ml) or chimeric CBTAU-22.1 (10 μg/ml). After thorough rinsing, goat-anti-mouse-EnVision+™ HRP-labelled secondary antibodies (DAKO) were incubated at RT for 30 min. DAB chromogen was used to visualize HRP-labeled structures. An additional microwave heating step in citrate buffer was performed after the DAB visualization for 10 min. This additional heating step removes all immune complexes, yet DAB precipitates remain. To block nonspecific binding of the secondary antibodies, sections were incubated with 2% normal rabbit serum (DAKO) at RT for 10 min before they were incubated with AT8 (Thermo Fisher, 0.25 μg/ml) or chimeric CBTAU-7.1 (2.5 μg/ml) at RT for 1 h. After washing, secondary biotinylated-rabbit-anti-mouse (DAKO) antibodies were allowed to bind for 45 min at RT. The sections were then incubated with alkaline phosphatase (AP) labeled streptavidin (Roche Applied Science) for 60 min. Liquid permanent red (LPR, DAKO) substrate was used to detect AP-labeled structures. Sections were counterstained with haematoxylin prior to permanent mounting in Aquatex (Merck, Darmstadt, Germany). Double labeling was analyzed and photographed by the use of a nuance multispectral imager (PerkinElmer, Waltham, MA, USA).

Free-floating (immuno) histochemistry: Formalin-fixed brain tissue was rinsed in distilled water and impregnated in increasing concentrations sucrose (10-20-30% in distilled water). When the tissue sank in the sucrose solution (after 2–4 days), it was transferred to the following sucrose concentration. After passing through the three concentrations the prepared material was stored at −20 °C until further use. Thirty µm-thick sections were transferred in wells of a 24-well culture plate (Sigma) and rinsed with PBS. Endogenous peroxidase activity was blocked by incubating the sections 30 min in PBS containing 0.3% H_2_O_2_. Subsequently sections were rinsed in washing buffer: 0.5% Tween20 (Sigma), 0.25% Triton X-100 (Merck), 0,1% gelatin from cold water fish skin (Sigma) and 1% bovine serum albumin (BSA, Roche) in PBS, and exposed to heat induced antigen retrieval using citrate buffer (pH 6.0) at 80 °C. After allowing the heated sections to regain RT, sections were rinsed in washing buffer and incubated with blocking buffer: 2% normal rabbit serum (Dako) in washing buffer. The blocking buffer was replaced by blocking buffer containing primary antibodies: CBTAU7.1 (5 µg/mL), CBTAU22.1 (5 µg/mL) and AT8 (0.25 µg/mL). The following day sections were thoroughly rinsed with washing buffer and incubated with HRP conjugated rabbit-anti-mouse antibody (DAKO) for 1 h. After extensive washing DAB was used as chromogen. Sections were counterstained with haematoxylin, dehydrated and mounted using Quick-D mounting medium.

For the gallyas silver staining 30 µm-thick sections were rinsed in distilled water and incubated in 5% periodic acid for 30 min at RT, followed by an incubation in silver iodide solution (4% sodium hydroxide, 10% potassium iodide and 0.35% silver nitrate in distilled water) for 30 min at RT. Subsequently, sections were washed in 0.5% acetic acid and developed with developer working solution (10 volumes 5% sodium carbonate solution, 3 volumes solution 0.2% ammonium nitrate, 0.2% silver nitrate and 1% Tungstosilicic acid solution, and 7 volumes 0.2% ammonium nitrate, 0.2% silver nitrate, 1% Tungstosilicic acid and 0.3% formaldehyde solution). After color development, sections were rinsed in 0.5% acetic acid, after which sections were incubated in 5% sodium thiosulphate and rinsed in distilled water. Stained sections were mounted on coated glass slides (Menzel-Gläser) and dried for at least 2 h at 37 °C. Subsequently, sections were fixed in 70% ethanol for 10 min, counterstained with hematoxylin, dehydrated, and mounted with Quick D mounting medium.

#### Data deposition

The atomic coordinates and structure factors of the CBTAU-7.1 Fab and CBTAU-22.1 Fab have been deposited in the Protein Data Bank, www.rcsb.org (PDB ID codes 5V7R and 5V7U).

## Results

**Fig. 1:**
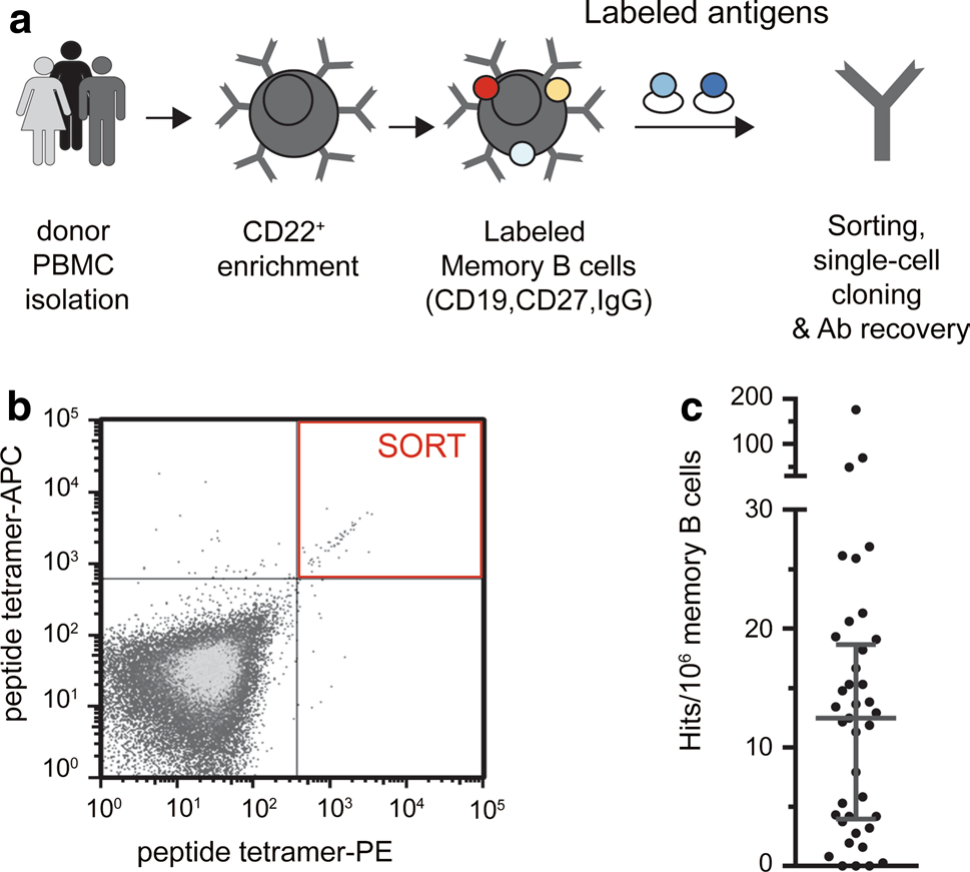
Recovery of naturally occurring monoclonal antibodies to pathogenic tau from asymptomatic individuals. **a** BSelex method used to recover tau-specific memory B cells. PBMCs were prepared from asymptomatic (non-AD) blood bank donors, and mature CD22^+^ B cells were positively selected with magnetic beads. Viable cells were stained with IgG-FITC, CD19-PerCPCy5.5, and CD27-PECy7, and with labeled peptide antigens and single-cell sorted on a Beckman Coulter MoFlo XDP. Antibody heavy and light variable chain sequences were recovered from single cells, cloned and expressed as full-length IgGs. **b** Polyvalent displays of tau phosphopeptides were labeled with APC or PE and gated on the double-positive population (PE^+^, APC^+^) to increase the signal-to-noise ratio. Memory B cells that showed reactivity to both APC- and PE-labeled peptides simultaneously were sorted as single cells into 96-well plates for further processing and recovery of IgH and IgL genes. Shown is a representative flow cytometric graph. **c** Estimated frequency of tau-specific B cells (Hits) per 10^6^ memory B cells interrogated in the different sorts. Based on sorting losses (gated cells not being deposited), cloning efficiency (ability to recover both antibody heavy and light chains from single cells), and reconfirmation rate (whether the cloned antibody showed tau binding), we estimate that on average there were 12.5 potential hits per 1x10^6^ CD22^+^ CD19^+^CD27^+^IgG^+^ memory B cells examined across all sorts. The *horizontal line* indicates median, and *error bars* the inter-quartile range

Blood samples from healthy donors between the ages of 18 and 66 years were screened for the presence of memory B cells reactive to tau using the BSelex method (Fig. 1a). Prior to sorting, B cells from individual donors’ PBMCs were enriched by CD22 positive selection and then tagged with fluorescently labeled CD19, CD27, and IgG antibodies to identify the peripheral IgG memory B cell population. After processing, we recovered 150,000 cells per donor sample on average, consistent with previous observations of about 0.05% of the PBMCs harvested [64]. Notwithstanding, based on these results, and to maximize the probability of identifying specific tau-reactive antibodies in the memory compartment, we increased the number of cells interrogated in each sort by pooling multiple (3 or 4) donor samples. We used a comprehensive panel of 122 tau peptides as antigenic probes. These peptides were synthesized, such that all well-characterized phosphorylation sites were represented, and when potential sites occurred within seven amino acids of each other, all combinations of singly and doubly phosphorylated peptides were included (Fig. S1). Since technical limitations prevented the use of the entire panel of tau phosphopeptides within each sort, we employed a strategy whereby varying subsets of peptides, up to 10 at a time, were selected for individual sorts in such a way that all peptides were eventually used in two or more sorts. As identification of rare specificities can easily be overwhelmed by poor separation of specific and nonspecific signals, we utilized polyvalent displays of tau peptide labeled with either APC or PE and gating on the double-positive population (PE^+^, APC^+^) to increase the signal-to-noise ratio and recover tau-specific B cells (Fig. 1b). Antibody heavy and light variable chain sequences were recovered from single cells, and full-length IgGs were cloned and expressed. In total, samples from 120 healthy donors were screened and 52 unique tau-binding antibodies were identified from the memory B cell donor pools. By back calculating, using the frequency of antigen-positive cells identified and taking into account sorting losses, cloning inefficiencies and reconfirmation rate, we estimate a median frequency of 12.5 potential hits per 1 × 10^6^ memory B cells examined across all sorts (Fig. 1c).

**Fig. 2:**
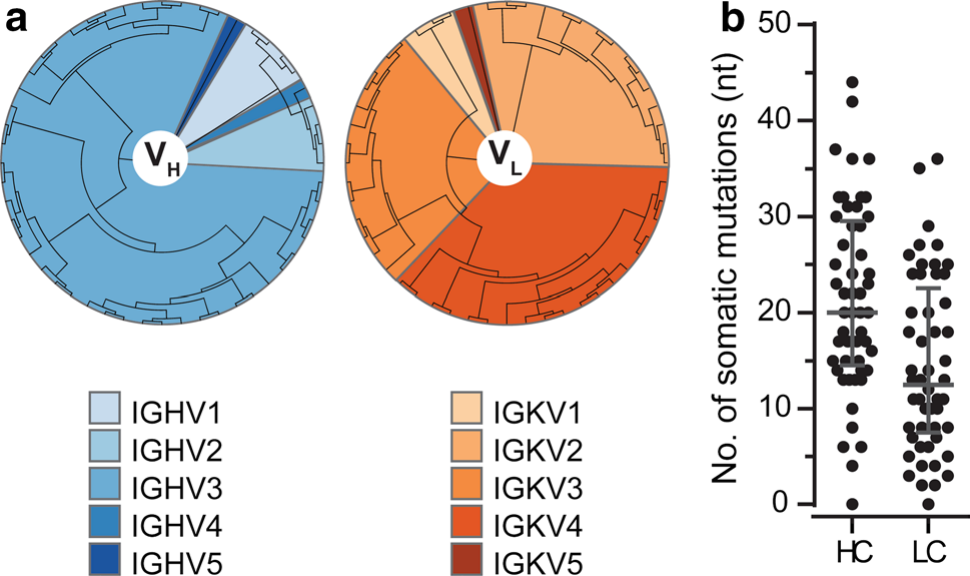
Sequence analysis of recovered anti-tau monoclonal antibodies. **a** Phylogenetic analysis of recovered tau antibody heavy and light chain variable regions was performed using the neighbor-joining algorithm (Jukes Cantor model) and illustrated as a circular tree. **b** Number of somatic mutations in V_H_ and V_L_ genes in 52 antibodies analyzed from IgG^+^ memory B cells with reactivity to tau. Mutations and identification of the closest germline were determined using IgBlast and IMGT databases. The *horizontal line* indicates median and *error bars* the inter-quartile range

Antibody sequences recovered from tau binders were analyzed for heavy and light chain germline usage and frequency of variable region somatic mutations. Sequence and germline usage information allowed assignment of the 52 tau antibodies to 35 unique clonal families (Fig. 2a, Table S1). Germline analysis showed a higher than expected preference (81%) for IGHV3, compared to a reported average of 35% of the naïve B cell population [20]. Primer sets used to clone antibody genes were previously confirmed to amplify all major heavy and light chain germline genes, so the abundance of VH3 heavy chain antibodies is unlikely to be the result of primer bias. Both heavy and light chain sequences revealed a number of somatic mutations, with median frequencies of 20 and 12.5 nucleotide substitutions for the heavy and light chain variable regions, respectively (Fig. 2b). The distribution of anti-tau antibodies into clonal families and the high degree of somatic mutations suggest that these antibodies share characteristics consistent with those evolving from ongoing antigen-driven responses.

Since memory B cell selections were performed using pools of peptides, an ELISA-based binding assay with each peptide was performed to identify the peptide-binding partner. For each antibody recovered, we were able to confirm binding to a single peptide from the initial bait pool, which provided an indication of its binding region (Table S1). Although we exploited the use of phosphopeptides to screen for tau-specific memory B cells, it remains possible that antibody binding can occur independently of phosphorylation. To assess whether antibody binding was dependent on peptide phosphorylation, hits were also screened against nonphosphopeptides spanning the corresponding regions. While half of the 52 antibodies recovered bound only to the phosphorylated cognate peptide, the other half reacted to both the phosphorylated and unphosphorylated peptides containing their epitopes (Table S1). Forty-one of the recovered antibodies recognize epitopes in the proline-rich (32 out of 52) and C-terminal (9 out of 52) domains, in accordance with the fact that—due to the distribution of phosphorylation sites on the tau protein—most of the peptides used as bait cover these regions (Fig. S1).

**Fig. 3:**
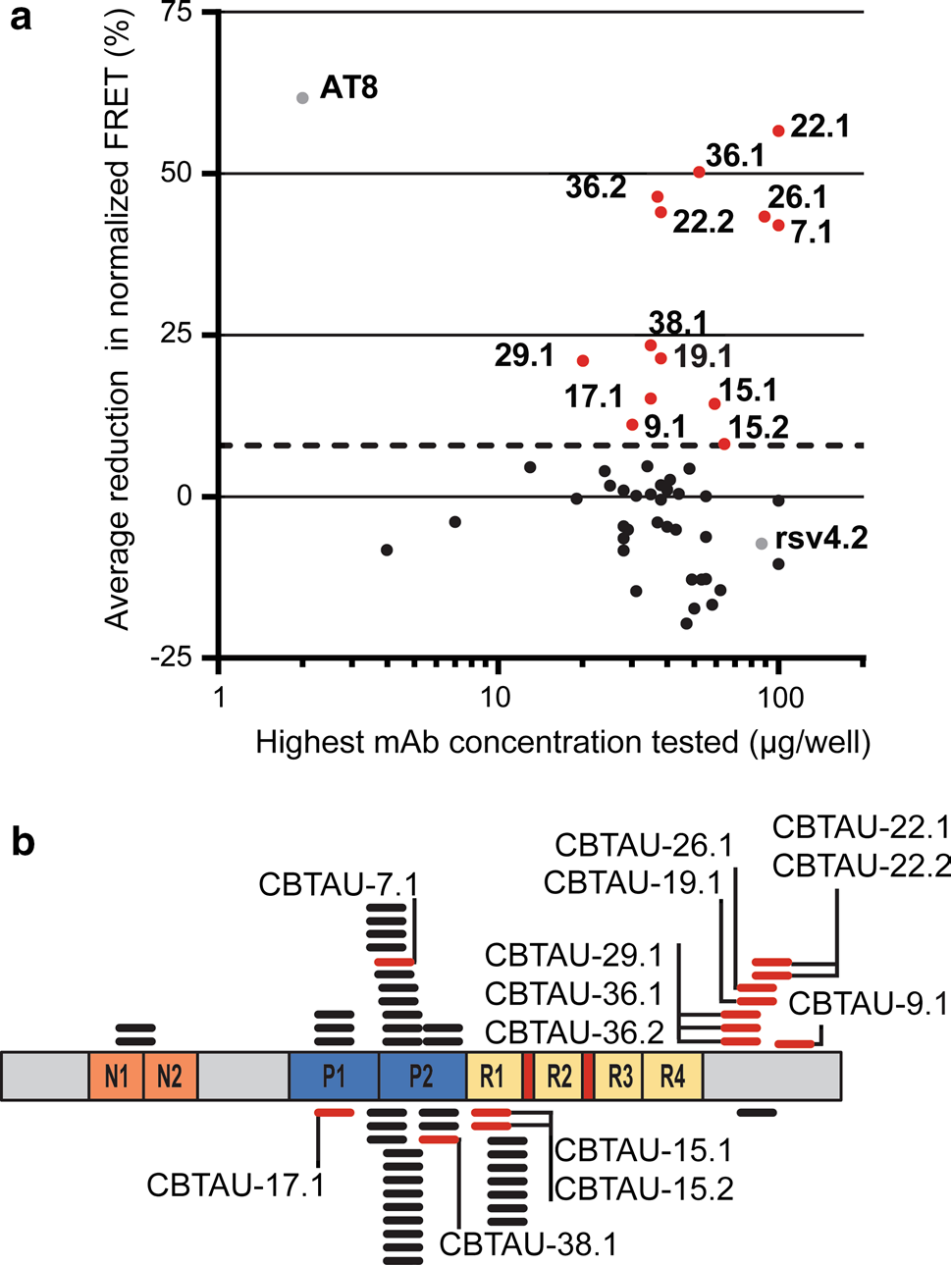
Antibody activity in an in vitro tau aggregation assay in relation to binding region and phospho-dependency. **a** Reduction in FRET signal of the highest tested concentration for each antibody. The names of antibodies that showed above-cutoff activity are indicated. Murine anti-tau antibody AT8 and anti-RSV-G antibody, RSV4.2, were included as positive and negative controls, respectively. *Dotted line* indicates a threshold (0% reduction + 2 × SD) below which measured activity is considered background. **b** Schematic showing the relative positions along tau isoform 2N4R of the tau peptides used to recover each of the 52 anti-tau antibodies. Highlighted (*red*) are the peptides recognized by those antibodies that show activity in the aggregation assay and the names of the respective antibodies are indicated. Cognate peptides of antibodies whose binding is dependent on phosphorylation are depicted above the representation of the tau isoform, while those of antibodies that bind to both phosphorylated and unphosphorylated peptides are depicted below the representation of the tau isoform. *N1* and *N2* indicate acidic inserts, *P1* and *P2* indicate proline-rich domains, and *R1*–*R4* indicate microtubule-binding repeat domains

We next assessed the ability of the antibodies to block tau aggregate seeding activity present in P301S spinal cord lysates. This cell-based biosensor assay was adapted from Yanamandra et al. and is based on the expression of the microtubule repeat domains of tau (aa 243–375) with a D280K mutation [65]. The repeat domains are fused either to yellow or cyan fluorescent protein and uptake of exogenous tau aggregates into the cells results in aggregation of the tau fusion proteins, which is detected by FRET. The PHF-tau-specific mouse antibody AT8 [7] and an anti-RSV-G specific antibody generated in house were used as positive and negative controls, respectively. For this qualitative assessment, we determined whether or not activity was detected at the highest tested concentration of each antibody. Since this concentration was dependent on the antibody expression of the batch produced, which varied among the different antibodies, there may be some false-negatives among antibodies that did not express well. Nevertheless, inhibitory activity was detected for 13 of the antibodies (Fig. 3a). Interestingly, this was the case for all (8 out of 8) phospho-dependent antibodies binding to the C-terminus of tau. In addition, some phospho-dependent as well as phospho-independent antibodies that bind in the center region of tau—the region containing the microtubule binding domains and forming the core of tau filaments—were also able to reduce tau aggregate seeding in vitro (Fig. 3b). Of note, none of the human antibodies showed more inhibitory activity than AT8, despite considerably higher concentrations tested (Fig. 3a). Titration of two of the antibodies, CBTAU-7.1 and CBTAU-22.1, confirmed the lower potency of the human antibodies in this assay (Fig. S2).

**Fig. 4:**
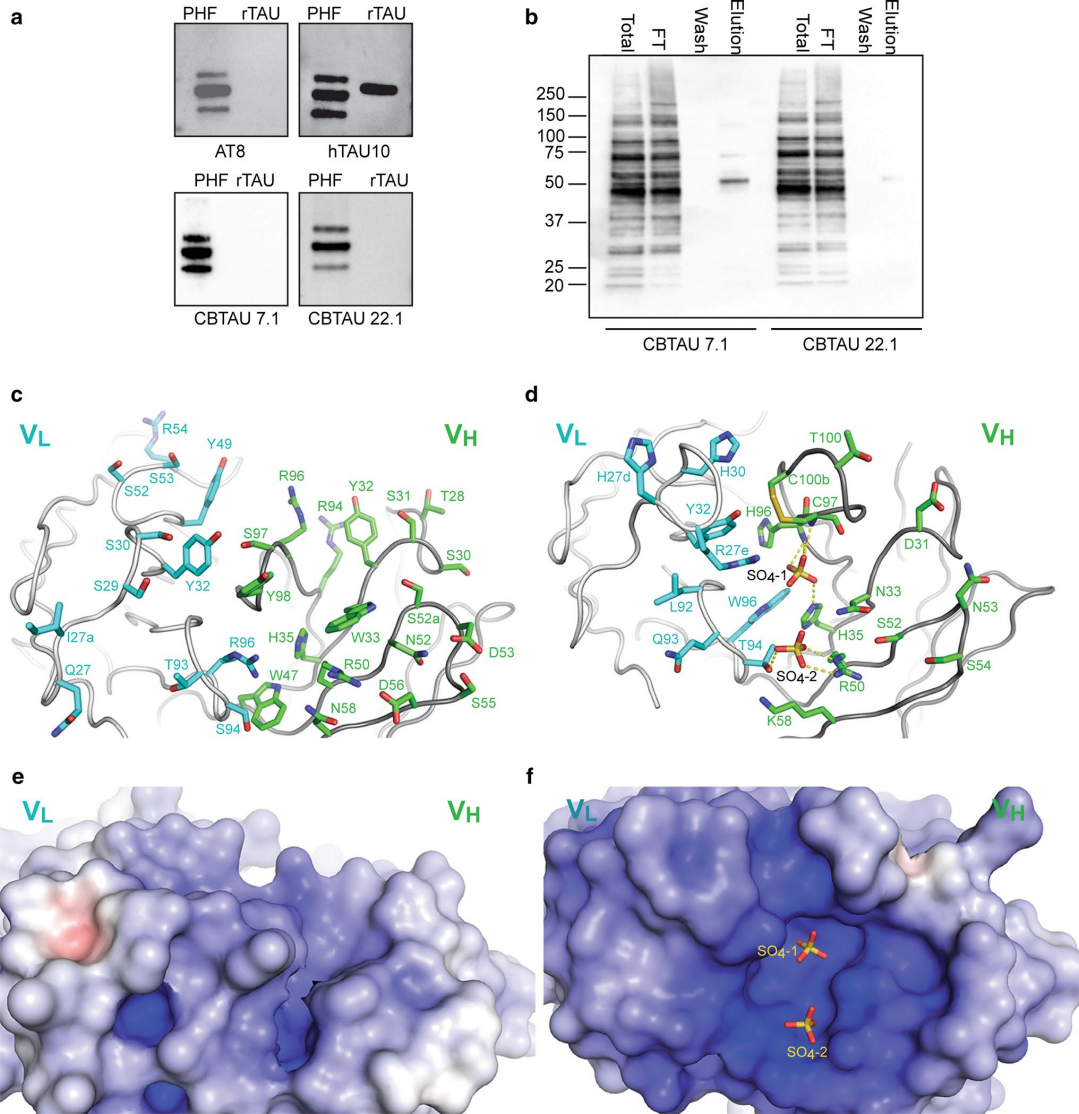
Specificity and structural characterization of human anti-tau antibodies. **a** Reactivity of AT8 (phospho-dependent), htau10 (non-phospho–dependent), CBTAU-7.1 and CBTAU-22.1 to PHF and recombinant human tau-441 by Western blot. Triple bands characteristic of PHF-tau correspond to approximately 68, 64, and 60 kDa. **b** Western blot of flow-through (FT), wash (Wash) and eluate (Elution) fractions of immunoprecipitation (IP) of P301S transgenic mice brainstem homogenate with CBTAU-7.1 (*left*) and CBTAU-22.1 (*right*), as well as untreated P301S (Total) homogenate. The blot was stained with total-Tau antibody HT7. Total volumes of all samples were kept similar to enable comparison of concentrations. (**c** and **d**) Crystal structures of the combining sites of CBTAU-7.1 and CBTAU-22.1 Fab, respectively. Some solvent-exposed CDR residues in and around the antibody combining site are shown in cyan carbon atoms (light chain) and green carbon atoms (heavy chain). Two sulfates were modeled in the combining site of CBTAU-22.1 (**d**). (**e** and **f**) Electrostatic potential surfaces in and around the combining sites of CBTAU-7.1 Fab and CBTAU-22.1 Fab, respectively. Electrostatic surface potentials were calculated using the APBS program [5]. Negatively charged regions are red, positively charged regions are blue, and neutral regions are white (−15 to 15 *K*_*b*_ *T*/*e*_*c*_ potential range). Both CBTAU-7.1 and CBTAU-22.1 Fab exhibit highly positively charged surfaces in their combining sites

As representatives for phospho-dependent antibodies showing activity in the in vitro tau aggregate seeding assay and binding to the proline-rich and the C-terminal domains, respectively, CBTAU-7.1 and CBTAU-22.1, were characterized further. The peptide reactivity observed using ELISA was extended by measuring binding to full-length recombinant tau (rTau) and PHF from human AD cortical tissue by Western blot. Consistent with previous reports, AT8 showed strong immunoreactivity to PHF (giving the characteristic three-band staining), but not to rTau (Fig. 4a), due to the absence of phosphorylation on the *E. coli* expressed protein. In contrast, the pan-tau antibody htau10 [46] recognized both PHF and rTau. Similar to AT8, CBTAU-7.1 and CBTAU-22.1 recognized PHFs, but no binding to rTau was observed, supporting the observation that binding of these antibodies requires phosphorylation of one or more residues within the epitope. Furthermore, immunoprecipitation experiments using brain samples from P301S mice confirmed the ability of CBTAU-7.1 and CBTAU-22.1 to bind tau under native conditions (Fig. 4b; Table S2).

To explore the interactions of the antibodies with tau, we mapped the epitopes using sets of differentially phosphorylated peptides. AT8 has previously been reported to recognize an epitope on tau between residues 194 and 212, phosphorylated at S202 and T205 [47] and also at S208, as reported more recently [39]. Our peptide mapping results confirmed the importance of phosphorylation of S202 and T205 for AT8 binding (Table S3). Antibody CBTAU-7.1 was mapped exclusively to the same peptide region as AT8 (Table S3), but its affinity for the doubly phosphorylated peptide at positions S202 and T205 was much lower than that of AT8 (5.8 µM vs 87.5 nM, Fig. S3). However, binding of CBTAU-7.1 appeared to be more promiscuous involving not only phospho-residues on positions S202 and T205, but also combinations of S198 + S202, S198 + T205, S199 + T205, and possibly Y197 + T205 (Table S3). That the epitopes to which CBTAU-7.1 and AT8 bind are similar but not identical is also clear from biolayer interferometry measurements, which confirmed that CBTAU-7.1 was able to bind to peptide phosphorylated at positions S198 and S202, whereas AT8 was not, and furthermore showed that phosphorylation of a third residue (S198), in addition to S202 and T205, increased binding by CBTAU-7.1 but had no effect on AT8 binding (Fig. S4).

Binding of CBTAU-22.1 was found to be dependent on phosphorylation of S422 (Table S3) and, as seen for CBTAU-7.1, affinity for phosphorylated peptide was modest (32.7 µM) (Fig. S2). Binding of both CBTAU-7.1 and CBTAU-22.1 was notably affected by ionic strength (Fig. S5), which suggests that binding is stabilized by ionic interactions with involvement of phospho groups in both cases. This notion is supported by crystal structures of CBTAU-7.1 and CBTAU-22.1 Fabs (Table S4) which we determined at 2.30 and 1.64 Å resolution, respectively. For CBTAU-7.1, the framework and all CDR loops can be fully modeled into the high-quality electron density map. The CBTAU-7.1 combining site is lined mostly by hydrophilic and aromatic residues (Fig. 4c). Basic residues are prevalent in and around the antibody binding site: Arg96 from light chain (Kabat numbering) and Arg50 from heavy chain are in the combining site and Arg94 and Arg96 from heavy chain line the rim of the presumed binding pocket (Fig. 4c). These positively charged residues create basic electrostatic pockets on the antibody surface (Fig. 4e) that could play an important role in the phosphopeptide recognition. In Fab CBTAU-22.1, the CDR loop residues also fit the electron density very well, except for the more flexible CDR L1 residues 27d, 27e, 28 and 29. A disulfide bond is formed in CDR H3 between Cys97 and Cys100b (Fig. 4d). Similar to CBTAU-7.1, three basic residues, Arg50 and Lys58 from the heavy chain, and Arg27e from the light chain, are present in the antibody combining site, which display very strong positive electrostatic potential (Fig. 4f). Interestingly, two sulfates from the crystallization buffer can be modeled into electron density in the highly basic combining site. One sulfate SO_4_-1 hydrogen bonds with His35 and the main-chain amides from His96 and Cys97 of heavy chain, with Arg27e in close proximity. Another sulfate SO_4_-2 forms a salt-bridge with Arg50 of heavy chain and hydrogen bonds with Thr94 of the light chain, with Lys58 of the heavy chain nearby. Furthermore, Lys58 and Arg27e are somatic mutations from the germline V-genes. Thus, these sulfate interactions and the strong basic nature of the combining site (Fig. 4f), likely explain the recognition of the phosphoryl groups in the tau phosphopeptides.

**Fig. 5:**
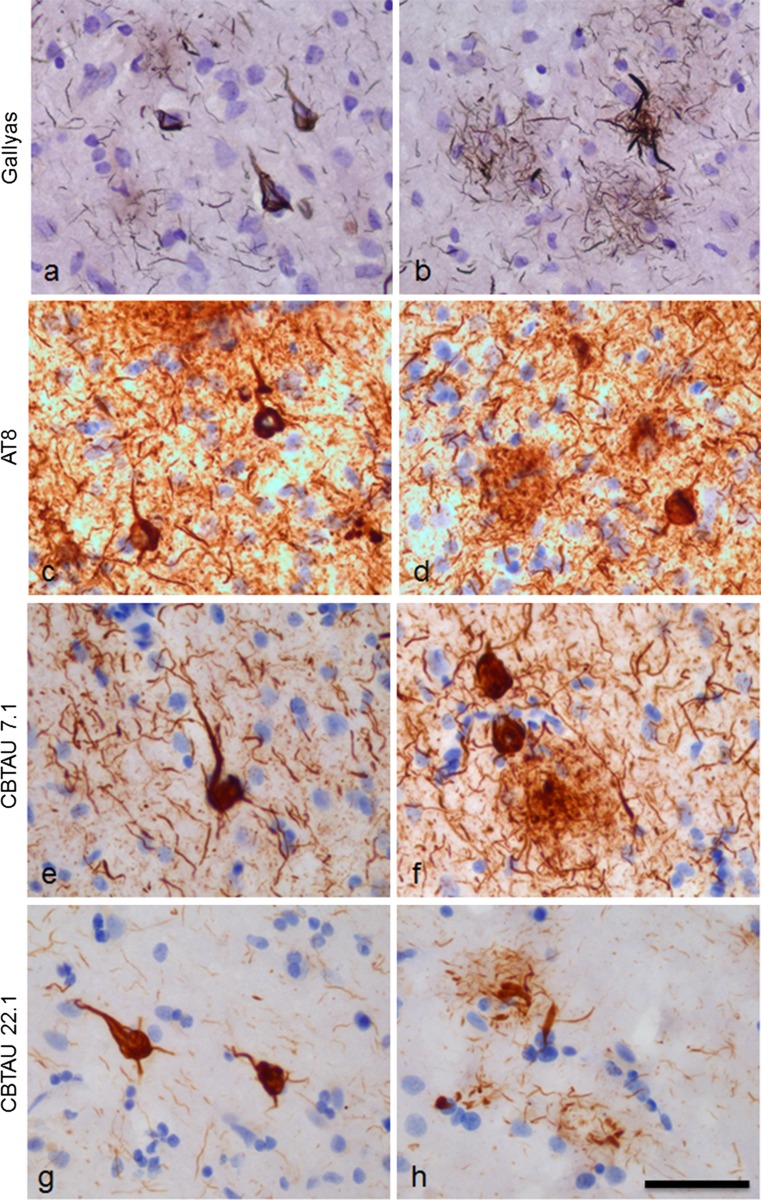
Overview of the pathological structures detected with Gallyas staining, AT8, CBTAU-7.1 and CBTAU-22.1. Staining was performed on 30 μm-thick, formalin-fixed, free-floating sections (temporal cortex, Alzheimer’s disease case, Braak VI). **a**, **b** Gallyas staining showing neurofibrillary changes. **c**, **d** AT8 immunostaining (0.25 μg/ml); **e**, **f** Immunostaining with CBTAU-7.1 (5 μg/ml). **g**, **b** Immunostaining with CBTAU-22.1 (5 μg/ml). Immunohistochemical detection with DAB (*brown*) and nuclei counterstained with haematoxylin (*blue*). *Bar* represents 50 μm

To further assess the specificity of the anti-tau antibodies, we conducted immunohistochemical staining on post-mortem brain tissue. CBTAU-7.1 and CBTAU-22.1 detected pathological tau structures in AD brain tissue, but not non-AD brain tissue. Comparable results were obtained when paraffin-embedded, formalin-fixed and fresh-frozen tissues were examined using either free-floating or slide-mounted immunohistochemistry and with or without antigen retrieval steps. Optimal immunohistochemical detection (i.e., staining of pathological tau without staining of the parenchyma) was obtained at 5 μg/ml for CBTAU-7.1 and CBTAU-22.1, whereas a concentration of 0.25 μg/ml was optimal for AT8 (Fig. S6). Observed immunoreactivity with CBTAU-7.1 and CBTAU-22.1 was compared with Gallyas staining for neurofibrillary changes and detection of p-tau using the AT8 antibody (Fig. 5). CBTAU-7.1 showed staining of neurofibrillary tangles, pretangles, neuritic plaques and neuropil threads that was comparable to AT8. CBTAU-22.1 also showed immunostaining of neurofibrillary tangles and neuritic plaques, but less neuropil threads compared to AT8 and CBTAU-7.1. Overall, the detection of p-tau with CBTAU-22.1 was comparable with the detection of neurofibrillary changes observed with Gallyas staining. Pretreatment of AD brain slides with alkaline phosphatase prohibited the detection of p-tau with CBTAU-7.1 and CBTAU-22.1 almost completely, confirming the high selectivity for phosphorylated epitopes on tau (Fig. S7).

**Fig. 6:**
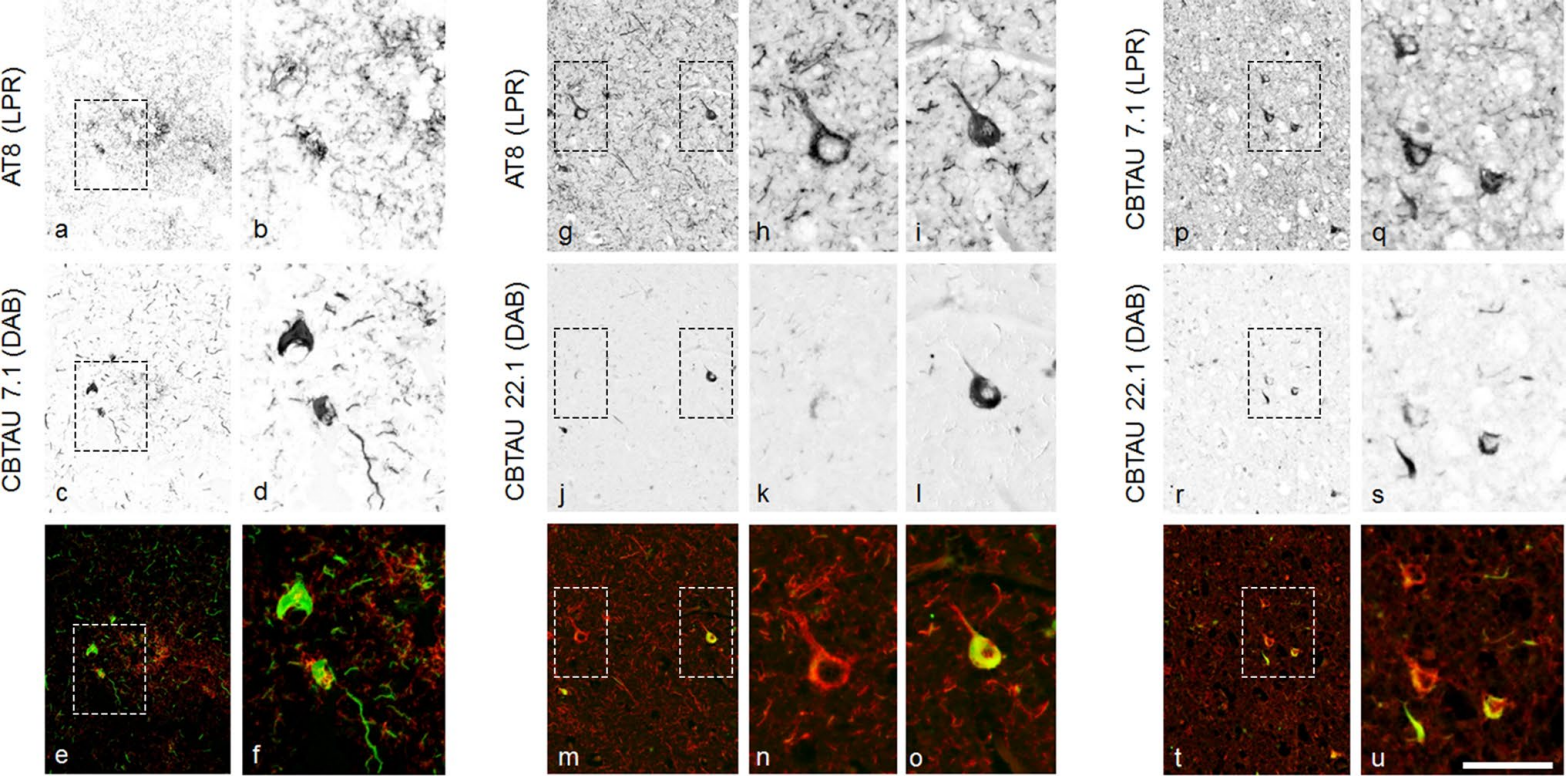
Double immunostaining with AT8, CBTAU-7.1 and CBTAU-22.1. Double immunostaining was performed on 5 μm-thick slide-mounted formalin-fixed, paraffin-embedded sections (temporal cortex, Alzheimer’s disease case, Braak VI). Double staining was analyzed with a spectral camera and digitally unmixed. Single staining patterns are presented separately (*gray scale*) and merged in artificial fluorescent colors. (**a**–**f**) Double immunostaining with AT8 (0.25 μg/ml, chromogen liquid permanent red, LPR) and CBTAU-7.1 (2.5 μg/ml, chromogen DAB). Areas indicated with *dotted lines* in **a**, **c**, and **e** are shown in higher magnification in **b**, **d**, and **f** respectively. (**g**–**o**) Double immunostaining with AT8 (0.25 μg/ml, LPR) and CBTAU-22.1 (10 μg/ml DAB). Areas indicated with *dotted lines* in **g**, **j**, and **m** are shown in higher magnification in **h/i**, **k/l**, and **n/o** respectively. (**p**–**u**); Double immunostaining with CBTAU-7.1 (2.5 μg/ml, LPR) and CBTAU-22.1 (10 μg/ml, DAB). Areas indicated with *dotted lines* in **p**, **r**, and **t** are shown in higher magnification in **q**, **s**, and **u** respectively. *Bar* represents 100 μm in overviews and 50 μm in magnifications

Immunostaining on temporal cortex and hippocampus slides of a cohort (*n* = 36) with different Braak stages ranging from I to VI showed that immunoreactivity of both CBTAU-7.1 and CBTAU-22.1 is Braak stage-dependent, similar to AT8 (Fig. S8). Neuronal immunostaining with the different antibodies was compared using double-immuno-labeling (Fig. 6). AT8 and CBTAU-7.1 showed co-labeling of individual neurons. However, a complete overlap in immunostaining was not observed (Fig. 6b, d, f). Using the co-immunolabeling protocol, the overall detection of tau structures with AT8 was reduced, suggesting that an incubation of CBTAU-7.1 in the first step of the co-immunolabeling experiment reduced subsequent immunodetection with AT8. CBTAU-22.1 immunostained fewer neurons compared to AT8. In addition to neurons that showed co-localization of AT8 and CBTAU-22.1 (Fig. 6i, l, o), neurons immunopositive for AT8 and negative for CBTAU-22.1 were observed (Fig. 6h, k, n). Co-labeling of CBTAU-7.1 and CBTAU-22.1 also showed partial overlap. Only some of the neurons that showed strong immunoreactivity with CBTAU-7.1 were detected with CBTAU-22.1 (Fig. 6o, s, u). Overall, CBTAU-22.1 showed partial overlap with AT8 and CBTAU-7.1 and was primarily confined to more dense or aggregated structures, again indicating that CBTAU-22.1 is more selective for IHC detection of neurofibrillary changes. Both CBTAU-7.1 and CBTAU-22.1 also show immunoreactivity of pathological tau structures in primary age-related tauopathy (PART) [13], progressive supranuclear palsy (PSP), frontotemporal dementia with microtubule-associated tau gene mutation (FTDP-17) and Pick’s disease cases, indicating that the phospho-epitopes detected by CBTAU-7.1 and CBTAU-22.1 are present throughout different tauopathies (Fig. S9). Consistent with our observations in AD brain tissue, CBTAU-7.1 showed a comparable immunostaining as observed with AT8, while CBTAU-22.1 showed immunostaining of dense pathological structures in different tauopathies.

## Discussion

Using a high-throughput, single-cell, screening methodology, we were able to recover a panel of 52 tau antibodies from the memory B cell compartment of healthy individuals between the ages of 18 and 66. Although we cannot distinguish whether antibodies recovered from the same pool of (up to four) donors arose from one or different donors based on the screening strategy we used, it would appear that a tau-reactive IgG response is not an uncommon feature in the healthy donor population screened. This result is in accordance with previous reports of the presence of naturally occurring antibodies to tau in sera of healthy individuals [29, 48, 54, 55]. A higher than expected proportion of the recovered antibodies was derived from the IGHV3 gene family. Similar heavy chain germline preferences have been previously reported for other targets. For example, a higher than expected proportion of influenza hemagglutinin stem binders appear to utilize the VH1-69 germline [12, 56, 58, 63], and this preference may reflect the ability of particular germline sequences to solve specific recognition problems [37, 38, 45].

The tau antibodies recognize a variety of epitopes across tau and binding of 26 of these antibodies is strictly dependent on the presence of phosphorylated residues in their respective epitopes. Thirteen antibodies showed activity in an in vitro aggregation assay and, although activity is not limited to antibodies that recognize phosphorylated tau, it is notable that all (eight of eight) phospho-dependent antibodies that bind to the C-terminus of tau show activity. Since anti-tau antibodies that block tau aggregate seeding in vitro have been shown to decrease pathology and improve cognition in a mouse model of AD [65], these results suggest that this region may be of particular interest as a target for therapy. Potential explanations for the apparent low potency of the set of human anti-tau mAbs compared to AT8 in the in vitro aggregation assay may be lower affinity for the seeds present in P301S spinal cord lysates or recognition of only a subset of these seeds, which may be caused by preferential recognition of additional post-translational modifications (e.g., acetylation), specific tau isoforms, or certain conformations of misfolded and aggregated tau. Further characterization of the antibodies should shed light on this issue. It would also be of interest to assess the ability of these human anti-tau antibodies to promote uptake of tau aggregates by microglia, which represents an Fc-mediated mechanism of action [19].

Two antibodies, CBTAU-7.1 and CBTAU-22.1, were studied in more detail as representatives for antibodies with activity in the in vitro tau aggregate seeding assay binding to distinct regions of tau. Both antibodies were considerably less potent in this assay than murine antibody AT8 (Fig. S2), which can likely be explained by a lower affinity for tau, as indicated by the relatively low affinity for their respective cognate peptides (Fig. S3). However, our Western blot and IP-MS data indicate that both antibodies are in fact directed against some species of tau and both appear to specifically stain tau structures in human brain by immunohistochemistry. Both CBTAU-7.1 and CBTAU-22.1 detect various pathological tau structures in AD brain tissue and selectively detect a phosphorylated epitope of tau in tissue. Crystal structures of both CBTAU-7.1 and CBTAU-22.1 revealed multiple positively charged arginine and lysine residues in the combining sites that are likely to be important for the recognition of the phosphate groups in the phosphorylated peptide epitopes in tau, as in other structurally characterized anti-tau antibodies such as pT231/pS235_1 with phosphorylated peptide pT231/pS235 [52], RB86 with phosphorylated peptide (416–430) [10] and AT8 with phosphorylated peptide (202–209) [40]. While CBTAU-7.1 binds the same region of tau as murine anti-PHF antibody AT8, it recognizes a slightly different epitope. In particular, CBTAU-7.1 appears to be more promiscuous with respect to the exact residues that need to be phosphorylated and may thus recognize a wider spectrum of phosphorylated tau species. However, CBTAU-7.1 shows similar detection of pathological structures compared to AT8 and follows a similar distribution throughout different Braak stages (Fig. S8). In contrast, CBTAU-22.1 immunostaining on AD brain tissue is confined to more dense or aggregated tau structures and shows comparable detection as obtained with the Gallyas staining of neurofibrillary changes. As the Gallyas staining is still the gold standard for the detection of neurofibrillary changes and neuropathological diagnosis of AD, it would be interesting to investigate the CBTAU-22.1 epitope for its specificity for the diagnosis of AD in a biomarker approach.

Our results suggest that ongoing antigen-driven immune responses to tau may not be an uncommon event within the donor population investigated. Whether these responses reflect changes that may predispose to, or protect against the development of Alzheimer’s disease remains to be determined. Either way, interrogating the human B cell repertoire for antibodies against modified (pathogenic) forms of tau can identify antibodies that may provide further insights into the underlying disease processes and potential intervention strategies. In particular, interrogating the memory B cell repertoire in human cohorts that are well defined in terms of age and health status (e.g., young vs elderly and healthy vs AD) would be of interest. For example, immuno-sequencing the antibody repertoires of such cohorts may lead to the identification of immune signatures that distinguish healthy individuals from individuals with (prodromal) AD. In addition, assays able to detect potential differences in antibody profiles (in either serum or CSF) may be able to distinguish healthy from AD. Given the range of anti-tau specificities found in the current study, such antibody assays should be able to measure the response against as many different tau epitopes as possible. Various studies have described the use of the presence of tau fragments in CSF [24, 31, 34, 53] and plasma [27, 30] as marker for the diagnosis of AD. Recent studies by Meredith et al. [44] and Barthélemy et al. [6] show that some fragments are significantly more abundant than others and that discrimination of AD from control is dependent on the subset of tau species measured [44]. However, current immunoassays are limited by a lack of available tau antibodies and measure only a limited range of tau species [6, 44]. Therefore, the presence of tau-specific antibodies in the human immune repertoire may provide a valuable source of antibodies that may be exploited for the development of antigen assays with increased resolution in terms of the tau species that can be measured thereby allowing for the development of more robust AD biomarkers. Finally, the diagnostic and predictive potential of any novel antibody- or antigen assay will need to be validated in cross-sectional and longitudinal cohort studies.

## Electronic supplementary material

Below is the link to the electronic supplementary material.

Supplementary material 1 (DOCX 24 kb)

Supplementary material 2 (DOCX 23 kb)

Supplementary material 3 (DOCX 20 kb)

Supplementary material 4 (DOCX 19 kb)

Supplementary material 5 (DOCX 21 kb)

Supplementary material 6 (TIFF 26520 kb)

Supplementary material 7 (TIFF 254 kb)

Supplementary material 8 (TIFF 523 kb)

Supplementary material 9 (TIFF 88 kb)

Supplementary material 10 (TIFF 90 kb)

Supplementary material 11 (TIFF 18857 kb)

Supplementary material 12 (TIFF 9863 kb)

Supplementary material 13 (TIFF 16385 kb)

Supplementary material 14 (TIFF 18488 kb)
