# Supplementary material for: Immunological memory to hyperphosphorylated tau in asymptomatic individuals
Source: Acta Neuropathol. 2017 Mar 24;133(5):767–83. doi: 10.1007/s00401-017-1705-y (PMC5390017; doi:10.1007/s00401-017-1705-y)
Supplement: Supplementary file 1 — Supplementary material 1 (DOCX 24 kb) [file 401_2017_1705_MOESM1_ESM.docx]

**Supporting Information**

**Immunological memory to hyperphosphorylated tau**

**in asymptomatic individuals**

Gabriel Pascual^a^, Jehangir S. Wadia^a,1^, Xueyong Zhu^b^, Elissa Keogh^a^, Başak Kükrer ^c^,

Jeroen van Ameijde^c^, Hanna Inganäs^c^, Berdien Siregar^c^, Gerrard Perdok^c^, Otto

Diefenbach^c^, Tariq Nahar^c^, Imke Sprengers^c^, Martin H. Koldijk^c^, Els C. Brinkman-van

der Linden^c^, Laura A. Peferoen^d^, Heng Zhang^b^, Wenli Yu^b^, Xinyi Li^a^, Michelle

Wagner^a,1^, Veronica Moreno^a,1^, Julie Kim^a^, Martha Costa^a^, Kiana West^e^, Zara Fulton^a,2^,

Lucy Chammas^a,3^, Nancy Luckashenak^a,4^, Lauren Fletcher^a^, Trevin Holland^a^, Carrie

Arnold^a^, R. Anthony Williamson^e^, Jeroen J. Hoozemans^d^, Adrian Apetri^c^, Frederique

Bard^a^, Ian A. Wilson^b,f^, Wouter Koudstaal^c,5^ & Jaap Goudsmit^c,g,h^

^a^ Janssen Prevention Center, Janssen Pharmaceutical Companies of Johnson & Johnson, 3210 Merryfield Row, San Diego, CA 92121, USA.

^b^ Department of Integrative Structural and Computational Biology, The Scripps Research Institute, La Jolla, CA 92037, USA.

^c^ Janssen Prevention Center, Janssen Pharmaceutical Companies of Johnson & Johnson, Archimedesweg 6, 2333 CN, Leiden, the Netherlands.

^d^ Department of Pathology, Amsterdam Neuroscience, VU University Medical Center, De Boelelaan 1117, 1081 HV, Amsterdam, the Netherlands.

^e^ Janssen Prevention Center, Janssen Pharmaceutical Companies of Johnson & Johnson, 2 Royal College Street, London, UK NW1 0NH.

^f^ Skaggs Institute for Chemical Biology, The Scripps Research Institute, La Jolla, CA 92037, USA.

^g^ Department of Neurology, Amsterdam Neuroscience, Academic Medical Center, Meidreefberg 9, 1105 AZ Amsterdam, the Netherlands.

^h^ Department of Epidemiology, Harvard T.H. Chan School of Public Health, 677 Huntington Avenue, Boston, MA 02115, USA.

^1^ Present address: Janssen R&D US, 3210 Merryfield Row, San Diego, CA 92121

^2^ Present address: Informa, Pharma intelligence, 3655 Nobel Drive, San Diego, CA 92122

^3^ Present address: TTP Labtech Inc, One Kendall Square, Cambridge, MA 02139-1594

^4^ Present address: Cidara Therapeutics, 6310 Nancy Ridge Drive, San Diego CA 92121

^5^ To whom correspondence should be addressed. E-mail: WKoudsta@its.jnj.com

**SI Figure Legends**

**Figure S1.** Schematic showing the relative position of the 122 tau phospho-peptides (black bars) used as baits, along tau isoform 2N4R. N1 and N2 indicate acidic inserts, P1 and P2 indicate proline-rich domains, and R1-R4 are the microtubule binding repeat domains. Phosphorylation sites are indicated as red dots.

**Figure S2. Dose-response of CBTAU-7.1, CBTAU-22.1 and AT8 in the *in vitro* tau aggregation assay.** Reduction in FRET signal of various concentrations of CBTAU-7.1, CBTAU-22.1 and murine anti-tau antibody AT8.

**Figure S3. Isothermal Titration Calorimetry (ITC) affinity measurements for CBTAU-7.1, AT8, and CBTAU-22.1 in complex with tau cognate peptides.** Variation in enthalpy is followed by incremental addition of mAb stock (140-200 μM) to tau peptide (30-40 μM). Continuous lines represent the best fit of experimental data assuming a single set of binding sites. Experiments were performed in PBS. Equilibrium dissociation constants are shown on the individual graphs. The sequences of peptides B1000 and V1088-5 are ^188^PPKSGDRSGYSSPG**S**PG**T**PGSR and ^404^SPRHLSNVSS**T**GSIDMVD**S**PQLATLA, respectively (phosphorylated residues in bold and underlined).

**Figure S4. Association and dissociation profiles for AT8 (top) and CBTAU-7.1 (bottom) and differentially phosphorylated tau peptides encompassing residues 188-209.** Association was followed by immersing the peptide-coated biosensor in solutions containing 100 nM monoclonal antibody. Dissociation was followed by immersing the biosensor in buffer without antibody. The buffer used for these experiments was obtained by diluting 10 fold the 10X ‘Pall ForteBio’s Kinetics Buffer’ in PBS. Phosphorylated residues in the different tau peptides are bold and underlined in the peptide sequences.

**Figure S5. Effect of ionic strength on the affinity of CBTAU-7.1 and CBTAU22.1.**  Association and dissociation kinetics for the interactions of CBTAU-7.1 with peptide V1091-5 (^192^GDRSGYSSPG**S**PG**T**PGSRSRT, top panel) and of CBTAU-22.1 with peptide V1088-23 (^406^RHLSNVSSTG**S**IDMVD**S**PQLATLA, bottom panel) at different ionic strengths. Association was followed by immersing the peptide-coated biosensors in solutions containing 100 nM monoclonal antibody. Dissociation was initiated by moving the biosensors to buffer without antibody. The buffer used for these experiments was obtained by diluting 10 fold the 10X ‘Pall ForteBio’s Kinetics Buffer’ in buffers of different ionic strengths and the composition of the different buffers are indicated.

**Figure S6.** Immunostaining with different concentrations of AT8, CBTAU-7.1, and CBTAU-22.1. Staining was performed on 5 μm thick slide-mounted formalin-fixed, paraffin-embedded sections using 5, 0.5 or 0.25 μg/ml antibody. Shown are representative images of the hippocampus (CA1/subiculum region) of an AD case. (**a-f**) Immunostaining with different dilutions of AT8. (**g-l**) immunostaining with different dilutions of CBTAU-7.1. (**m-f**) Immunostaining with different dilutions of CBTAU 22.1. Immunohistochemical detection with DAB (brown) and nuclei were counterstained with haematoxylin (blue). Bar represents 200 μm in **a-c**, **g-i**, **m-o** and 50 μm in **d-f**, **j-l**, **p-r**.

**Figure S7.** Inhibition of immunostaining with AT8, CBTAU-7.1, and CBTAU-22.1 after alkaline phosphatase pretreatment. Immunohistochemical detection was done on 5 μm thick slide-mounted, formalin-fixed, paraffin-embedded hippocampus. Immunoreactivity observed with 0.25 μg/ml AT8 without (**a, b**) and with (**c, d**) alkaline phosphatase pretreatment. Immunoreactivity observed with 5 μg/ml CBTAU-7.1 without (**e, f**) and with (**g, h**) alkaline phosphatase pretreatment. Immunoreactivity observed with 5 μg/ml CBTAU-22.1 without (**i, j**) and with (**k, l**) alkaline phosphatase pretreatment. Immunohistochemical detection with DAB (brown) and counterstaining with haematoxylin (blue). Bar represents 200 μm in **a, c, d, e, g, i** and **k**; and 50 μm in **b, d, f, h, j**, and **l**.

**Figure S8.** Immunostaining of 5 μm thick slide-mounted, formalin-fixed, paraffin-embedded hippocampal (CA1/subiculum) sections derived from cases with different Braak stages. Immunohistochemical detection was done on formalin-fixed paraffin embedded brain tissue with AT8 (0.25 μg/ml), CBTAU-7.1 (2.5 μg/ml) and CBTAU-22.1 (10 μg/ml). Immunohistochemical detection with DAB (brown) and counterstaining with haematoxylin (blue). Bar represents 200 μm.

**Figure S9.** Overview of pathological tau structures detected in various tauopathies with AT8, CBTAU-7.1 and CBTAU-22.1. The immunoreactive staining pattern of CBTAU-7.1 and CBTAU-22.1 was assessed on 5µm thick parrafin-embedded post-mortem brain tissue sections of primary age-related tauopathy (PART, n=3), progressive supranuclear palsy (PSP, n=3), frontotemporal dementia with microtubule-associated tau gene mutation (FTDP-17, n=3) and Pick’s disease (n=3). The immunoreactive staining patterns of CBTAU-7.1 (2,5µg/ml) and CBTAU-22.1 (10µg/ml) were compared to the immunoreactivity observed with AT8 antibody (0,25µg/ml). In the hippocampus of PART cases, neurofibrillary tangles and neuropil threads were observed with AT8 and CBTAU-7.1 (**a, b**). With CBTAU-22.1, neurofibrillary tangles were observed in this brain region, however, compared to AT8 and CBTAU-7.1 less neuropil threads were observed (**c**). Examination of the caudate nucleus of PSP cases showed presence of immunoreactivity in neurons, neurites and astrocytes using the AT8 and CBTAU-7.1 antibodies (**d, e**). Clearly less immunoreactivity was observed with the CBTAU-22.1 antibody on PSP caudate nucleus (**f**). In FTDP-17, AT8 and CBTAU-7.1 revealed immunoreactive neuronal inclusions and neuropil threads, while CBTAU-22.1 showed dense neuronal inclusions in absence of immunoreactive neurites (**g-i**). In the frontal cortex of cases with Pick’s disease AT8 and CBTAU-7.1 showed immunoreactive astrocytes, which were hard to detect with CBTAU-22.1 (J-L). Neuropil threads and Pick bodies were easily recognized with AT8 and CBTAU-7.1 immunostaining (**m, n**). CBTAU-22.1 showed immunoreactivity of Pick bodies in the absence of positive neuropil threads (**o**). Binding of the antibodies was vizualised by a HRP-DAB interaction (brown) and nuclei were counterstained with haematoxylin (blue). The pictures show the charachteristic pathological tau structures observed in the different tauopathies shown. For each tauopathy three different cases were analysed and representative pictures are presented. Scale bar represents 25µm.
