# Supplementary material for: Immunological memory to hyperphosphorylated tau in asymptomatic individuals
Source: Acta Neuropathol. 2017 Mar 24;133(5):767–83. doi: 10.1007/s00401-017-1705-y (PMC5390017; doi:10.1007/s00401-017-1705-y)
Supplement: Supplementary file 2 — Supplementary material 2 (DOCX 23 kb) [file 401_2017_1705_MOESM2_ESM.docx]

**Table S1. Characteristics of antibody panel.**

| CBTAU | IGHV | IGKV | Somatic mutations  heavy chain ^a^ | | Somatic mutations  light chain ^a^ | | Binding region  cognate peptide ^b^ | Phospho-specific |
| --- | --- | --- | --- | --- | --- | --- | --- | --- |
|  |  |  | NT | AA | NT | AA |  |  |
| 1.1 | 3-7 | 3-15 | 13 | 10 | 5 | 4 | 204-221 | N |
| 1.2 | 3-7 | 3-15 | 20 | 9 | 17 | 13 | 204-221 | N |
| 1.3 | 3-7 | 3-15 | 17 | 10 | 20 | 10 | 204-221 | N |
| 1.4 | 3-7 | 3-15 | 17 | 10 | 12 | 10 | 204-221 | N |
| 2.1 | 3-7 | 1D-33 | 8 | 7 | 7 | 5 | 204-221 | N |
| 3.1 | 3-23 | 4-1 | 18 | 11 | 10 | 7 | 386-409 | N |
| 4.1 | 3-15 | 4-1 | 36 | 18 | 26 | 14 | 204-221 | Y |
| 5.1 | 3-23 | 3-15 | 23 | 14 | 18 | 13 | 194-212 | N |
| 7.1 | 3-74 | 3-20 | 15 | 6 | 11 | 7 | 194-212 | Y |
| 8.1 | 3-33 | 4-1 | 16 | 9 | 4 | 2 | 194-212 | Y |
| 9.1 | 3-15 | 2-30 | 17 | 10 | 5 | 4 | 414-430 | Y |
| 10.1 | 3-73 | 4-1 | 29 | 20 | 25 | 14 | 257-272 | N |
| 10.2 | 3-73 | 4-1 | 36 | 22 | 29 | 14 | 257-272 | N |
| 10.3 | 3-73 | 4-1 | 32 | 21 | 27 | 15 | 257-272 | N |
| 10.4 | 3-73 | 4-1 | 30 | 19 | 24 | 13 | 257-272 | N |
| 10.5 | 3-73 | 4-1 | 32 | 20 | 25 | 14 | 257-272 | N |
| 10.6 | 3-73 | 4-1 | 30 | 19 | 25 | 13 | 257-272 | N |
| 10.7 | 3-73 | 4-1 | 31 | 18 | 27 | 16 | 257-272 | N |
| 11.1 | 3-7 | 3-15 | 4 | 2 | 3 | 1 | 204-221 | N |
| 11.2 | 3-7 | 3-15 | 15 | 8 | 13 | 11 | 204-221 | N |
| 12.1 | 3-7 | 3-15 | 13 | 7 | 11 | 7 | 204-221 | N |
| 13.1 | 3-23 | 3D-15 | 42 | 16 | 36 | 22 | 194-212 | N |
| 13.2 | 3-23 | 3D-15 | 44 | 19 | 35 | 22 | 194-212 | N |
| 14.1 | 3-48 | 2-30 | 20 | 14 | 2 | 0 | 189-206 | Y |
| 15.1 | 3-49 | 2-30 | 22 | 16 | 18 | 8 | 248-265 | N |
| 15.2 | 3-49 | 2-30 | 26 | 17 | 21 | 8 | 248-265 | N |
| 16.1 | 3-7 | 3-15 | 13 | 9 | 8 | 4 | 204-221 | N |
| 17.1 | 2-5 | 2D-29 | 31 | 13 | 24 | 11 | 165-182 | N |
| 18.1 | 4-61 | 4-1 | 10 | 7 | 4 | 2 | 200-219 | Y |
| 19.1 | 3-53 | 2-30 | 29 | 16 | 14 | 11 | 394-416 | Y |
| 20.1 | 3-23 | 4-1 | 18 | 11 | 8 | 4 | 61-78 | Y |
| 22.1 | 1-2 | 2-28 | 24 | 18 | 10 | 9 | 406-429 | Y |
| 22.2 | 1-2 | 2-28 | 23 | 17 | 10 | 9 | 406-429 | Y |
| 23.1 | 3-48 | 2-30 | 37 | 23 | 8 | 5 | 187-206 | Y |
| 23.2 | 3-48 | 2-30 | 32 | 20 | 7 | 4 | 187-206 | Y |
| 24.1 | 1-2 | 4-1 | 22 | 12 | 11 | 6 | 228-245 | N |
| 26.1 | 3-72 | 1-16 | 15 | 10 | 8 | 5 | 399-421 | Y |
| 29.1 | 3-21 | 5-45 ^c^ | 24 | 12 | 24 | 13 | 386-409 | Y |
| 30.1 | 2-5 | 2-24 | 27 | 15 | 24 | 15 | 221-245 | N |
| 31.1 | 3-48 | 2-30 | 32 | 21 | 14 | 8 | 165-182, 189-206 ^d^ | Y |
| 32.1 | 2-5 | 4-1 | 20 | 11 | 18 | 12 | 228-245 | Y |
| 32.2 | 2-5 | 4-1 | 13 | 7 | 20 | 12 | 228-245 | Y |
| 33.1 | 3-49 | 4-1 | 6 | 5 | 3 | 3 | 200-219 | Y |
| 34.1 | 5-51 | 3-20 | 6 | 5 | 6 | 6 | 204-221 | Y |
| 35.1 | 3-7 | 3-15 | 14 | 9 | 6 | 3 | 204-221 | Y |
| 36.1 | 3-11 | 4-1 | 14 | 7 | 13 | 10 | 414-430 | Y |
| 36.2 | 3-11 | 4-1 | 17 | 8 | 13 | 10 | 414-430 | Y |
| 37.1 | 3-48 | 2-30 | 20 | 14 | 11 | 8 | 165-182 | Y |
| 37.2 | 3-48 | 2-30 | 25 | 14 | 15 | 9 | 165-182 | Y |
| 38.1 | 1-2 | 4-1 | 22 | 12 | 11 | 6 | 221-245 | N |
| 39.1 | 3-30 | 1-17 | 0 | 0 | 0 | 0 | 59-78 | Y |
| 40.1 | 3-48 | 2-30 | 14 | 0 | 2 | 1 | 165-182 | Y |

^a^ V-gene as defined in IMGT database

^b^ Numbering according to the longest human tau isoform containing 441 residues

^c^ Lambda instead of kappa light chain

^d^ Antibody shows reactivity to two non-overlapping peptides
