# Supplementary material for: Immunological memory to hyperphosphorylated tau in asymptomatic individuals
Source: Acta Neuropathol. 2017 Mar 24;133(5):767–83. doi: 10.1007/s00401-017-1705-y (PMC5390017; doi:10.1007/s00401-017-1705-y)
Supplement: Supplementary file 3 — Supplementary material 3 (DOCX 20 kb) [file 401_2017_1705_MOESM3_ESM.docx]

**Table S2. Tau peptides identified by mass spectrometry.** Proteins from P301S brain lysates immunoprecipitated by CBTAU-7.1 and CBTAU-22.1 were eluted with 1% aqueous formic acid. Eluents were evaporated and reconstituted in PBS yielding samples directly amenable to trypsin digestion followed by LC-MS/MS analysis. MS results from the eluates indicate that these IP-enriched samples contain tau protein, confirming that the bands seen on the Western blot are indeed tau.

| Residue | Tau peptide | *m/z* | Charge | | CBTAU-7.1  Intensity | CBTAU-22.1  Intensity |
| --- | --- | --- | --- | --- | --- | --- |
| 151-155 | IATPR | 279.2  557.3 | | 2+  1+ | 2.0E4  3.1E3 | 3.6E3  8.1E2 |
| 156-163 | GAAPPGQK | 363.2  725.4 | | 2+  1+ | 3.1E3  4.1E2 | ND |
| 175-180 | TPPAPK | 305.6  610.3 | | 2+  1+ | 7.0E3  1.9E3 | 1.1E3  3.5E2 |
| 195-209 | SGYSSPGSPGTPGSR | 697.3 | | 2+ | 2.7E3 | ND |
| 212-224 | TPSLPTPPTREPK | 474.3  710.9 | | 3+  2+ | 1.2E4  1.3E3 | ND |
| 225-230 | KVAVVR | 336.2  671.5 | | 2+  1+ | 1.1E4  8.4E3 | ND |
| 226-230 | VAVVR | 272.2  543.4 | | 2+  1+ | 8.0E3  1.8E3 | 2.1E3  5.4E2 |
| 260-267 | IGSTENLK | 431.2  861.5 | | 2+  1+ | 3.3E4  2.3E3 | 4.6E3  3.3E2 |
| 275-280 | VQIINK | 357.7  714.5 | | 2+  1+ | 4.3E4  3.5E3 | 6.7E3  5.8E2 |
| 281-290 | KLDLSNVQSK | 377.9  566.3 | | 3+  2+ | 4.2E4  1.9E4 | 6.9E3  2.2E3 |
| 344-349 | LDFKDR | 397.2  793.4 | | 2+  1+ | 8.3E3  7.3E2 | 1.4E3  2.2E2 |
| 354-369 | IGSLDNITHVPGGGNK | 526.95  789.9 | | 3+  2+ | 4.6E4  6.0E3 | 4.9E3  7.2E2 |
| 376-379 | LTFR | 268.7  536.3 | | 2+  1+ | 3.2E4  4.8E3 | 6.5E3  7.4E2 |
| 396-406 | SPVVSGDTSPR | 551.3  1101.6 | | 2+  1+ | 5.0E3  2.8E2 | ND |

ND: Not detected
