# Supplementary material for: Immunological memory to hyperphosphorylated tau in asymptomatic individuals
Source: Acta Neuropathol. 2017 Mar 24;133(5):767–83. doi: 10.1007/s00401-017-1705-y (PMC5390017; doi:10.1007/s00401-017-1705-y)
Supplement: Supplementary file 4 — Supplementary material 4 (DOCX 19 kb) [file 401_2017_1705_MOESM4_ESM.docx]

**Table S3. Epitope mapping using differentially phosphorylated peptides.** Peptides were captured onto streptavidin-coated plates and antibody binding was determined by ELISA and scored as weak (+), moderate (++), strong (+++), or no binding (N). Phosphorylated residues are indicated in red.

| AT8 | | |
| --- | --- | --- |
| **Peptide** | **Sequence** | **Binding** |
| 189-212 | P K S G D R S G Y S S P G S P G T P G S R S R T | N |
| 192-211 | G D R S G Y S S P G S P G T P G S R S R | N |
| 192-212 | G D R S G Y S S P G S P G T P G S R S R T | N |
| 192-209 | G D R S G Y S S P G S P G T P G S R | +++ |
| 192-215 | G D R S G Y S S P G S P G T P G S R S R T P S L | N |
| 192-217 | G D R S G Y S S P G S P G T P G S R S R T P S L P T | N |
| 194-212 | R S G Y S S P G S P G T P G S R S R T | +++ |
| 194-212 | R S G Y S S P G S P G T P G S R S R T | N |
| 195-212 | S G Y S S P G S P G T P G S R S R T | N |
| CBTAU-7.1 | | |
| **Peptide** | **Sequence** | **Binding** |
| 186-218 | G E P P K S G D R S G Y S S P G S P G T P G S R S R T P S L P T P | N |
| 187-212 | E P P K S G D R S G Y S S P G S P G T P G S R S R T | + |
| 188-205 | P P K S G D R S G Y S S P G S P G T | N |
| 188-206 | P P K S G D R S G Y S S P G S P G T P | N |
| 188-209 | P P K S G D R S G Y S S P G S P G T P G S R | ++ |
| 188-212 | P P K S G D R S G Y S S P G S P G T P G S R S R T | + |
| 189-206 | P K S G D R S G Y S S P G S P G T P | N |
| 189-209 | P K S G D R S G Y S S P G S P G T P G S R | N |
| 189-212 | P K S G D R S G Y S S P G S P G T P G S R S R T | + |
| 190-209 | K S G D R S G Y S S P G S P G T P G S R | N |
| 192-209 | G D R S G Y S S P G S P G T P G S R | N |
| 192-215 | G D R S G Y S S P G S P G T P G S R S R T P S L | N |
| 192-217 | G D R S G Y S S P G S P G T P G S R S R T P S L P T | N |
| 194-212 | R S G Y S S P G S P G T P G S R S R T | + |
| 194-212 | ­­R S G Y S S P G S P G T P G S R S R T | N |
| CBTAU-22.1 | | |
| **Peptide** | **Sequence** | **Binding** |
| 404-421 | S P R H L S N V S S T G S I D M V D | N |
| 404-429 | S P R H L S N V S S T G S I D M V D S P Q L A T L A | +++ |
| 405-423 | P R H L S N V S S T G S I D M V D S P | N |
| 406-423 | P R H L S N V S S T G S I D M V D S P | N |
| 406-429 | R H L S N V S S T G S I D M V D S P Q L A T L A | +++ |
| 409-428 | S N V S S T G S I D M V D S P Q L A T L | N |
| 412-429 | S S T G S I D M V D S P Q L A T L A | +++ |
| 412-434 | S S T G S I D M V D S P Q L A T L A D E V S A | +++ |
