# Supplementary material for: Immunological memory to hyperphosphorylated tau in asymptomatic individuals
Source: Acta Neuropathol. 2017 Mar 24;133(5):767–83. doi: 10.1007/s00401-017-1705-y (PMC5390017; doi:10.1007/s00401-017-1705-y)
Supplement: Supplementary file 5 — Supplementary material 5 (DOCX 21 kb) [file 401_2017_1705_MOESM5_ESM.docx]

**Table S4.** **Data collection and refinement statistics for human anti-tau Fabs**

| Data set | CBTAU-7.1 | CBTAU-22.1 |
| --- | --- | --- |
| Space group | P4_1_ | P2_1_ |
| Unit cell (Å) | *a* = *b* = 83.9,  *c* = 109.4 | *a* = 52.5,  *b* = 67.2,  *c* = 62.5 |
| *β* angle (deg.) |  | *β* = 94.4 |
| Resolution (Å) ^a^ | 50.0-2.30  (2.34-2.30) | 50.0-1.64  (1.67-1.64) |
| X-ray source | ALS 5.0.3 | APS 23ID-B |
| Unique reflections | 33,665 | 53,078 |
| Redundancy ^a^ | 7.1 (6.4) | 3.7 (3.5) |
| Average I/σ(I) ^a^ | 52.1 (2.9) | 23.7 (1.7) |
| Completeness ^a^ | 99.9 (100) | 99.9 (100) |
| *R*_sym_^a,b^ | 0.07 (0.79) | 0.08 (0.66) |
| *R*_pim_^a,b^ | 0.03 (0.34) | 0.05 (0.41) |
| CC_1/2_ ^a,c^ | 0.999 (0.821) | 0.995 (0.684) |
| Fabs in a.u. | 1 | 1 |
| *V*_m_ (Å^3^/Da) | 3.9 | 2.2 |
| Reflections (work/free) | 31,904/1,704 | 50,354/2,696 |
| *R*_cryst_^d^ | 0.171 | 0.176 |
| *R*_free_^e^ | 0.224 | 0.217 |
| Refined atoms  Fab  Waters | 3,313  87 | 3,351  462 |
| *B*-values (Å^2^)  Fab  Waters  Sulfates | 71  61  - | 25  37  26 |
| Wilson *B*-values (Å^2^) | 62 | 20 |
| R.m.s.d. bond (Å) | 0.009 | 0.007 |
| R.m.s.d. angle (deg.) | 1.23 | 1.21 |
| Ramachandran values (%)^f^ | 95.9, 0.2 | 97.7, 0.5 |
| PDB codes | 5V7R | 5V7U |

a.u., asymmetric unit.

^a^ Parentheses denote outer-shell statistics.

^b^ *R*_sym_ = ∑*_hkl_*∑*_i_* |*I_hkl,i_* - <*I_hkl_*>| /∑*_hkl_*∑*_i_* *I_hkl,i_* and *R*_pim_ = ∑*_hkl_*[1/(*N*-1)]^1/2^∑*_i_* |*I_hkl,i_* - <*I_hkl_*>| /∑*_hkl_*∑*_i_* *I_hkl,i_*, where *I_hkl,i_* is the scaled intensity of the i^th^ measurement of reflection *h*, *k*, *l*, < *I_hkl_*> is the average intensity for that reflection, and *N* is the redundancy.

^c^ CC_1/2_= Pearson’s correlation coefficient between random half datasets. CC_1/2_= ρ_X,Y_ = cov(X,Y)/σ_x_σ_y_ = E[(X-μ_x_)(Y-μ_y_)]/σ_x_σ_y_.

^d^ *R*_cryst_ = ∑*_hkl_* |*F_o_* - *F_c_*| / ∑*_hkl_* |*F_o_*|, where *F_o_* and *F_c_* are the observed and calculated structure factors.

^e^ *R*_free_ was calculated as for *R*_cryst_, but on 5% of data excluded before refinement.

^f^ The values are percentage of residues in the favored and outliers regions analyzed by MolProbity.
