# Supplementary figures and images for: Immunological memory to hyperphosphorylated tau in asymptomatic individuals
Source: Acta Neuropathol. 2017 Mar 24;133(5):767–83. doi: 10.1007/s00401-017-1705-y (PMC5390017; doi:10.1007/s00401-017-1705-y)

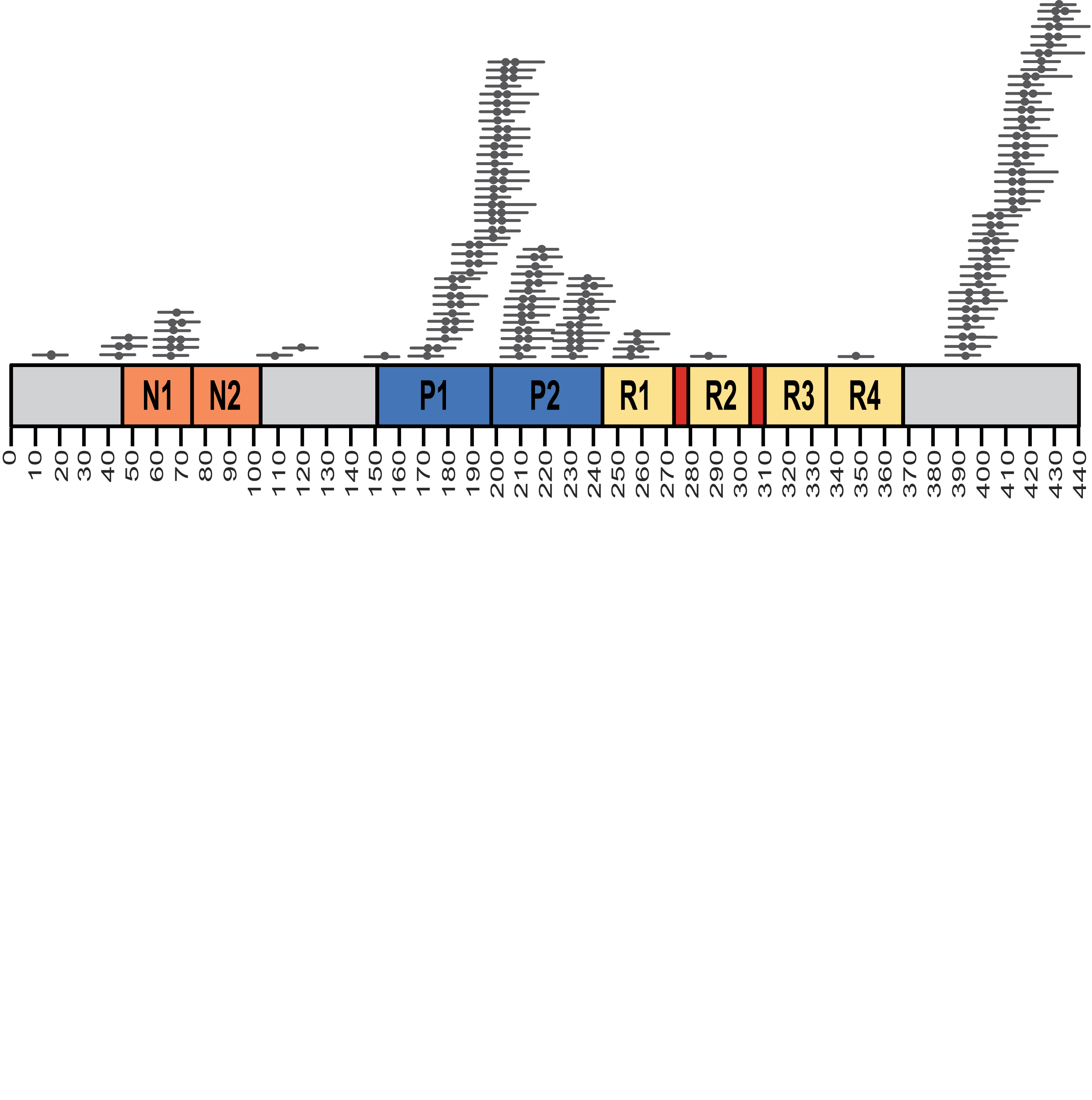

Supplement: Supplementary file 6 — Supplementary material 6 (TIFF 26520 kb) [file 401_2017_1705_MOESM6_ESM.tif]

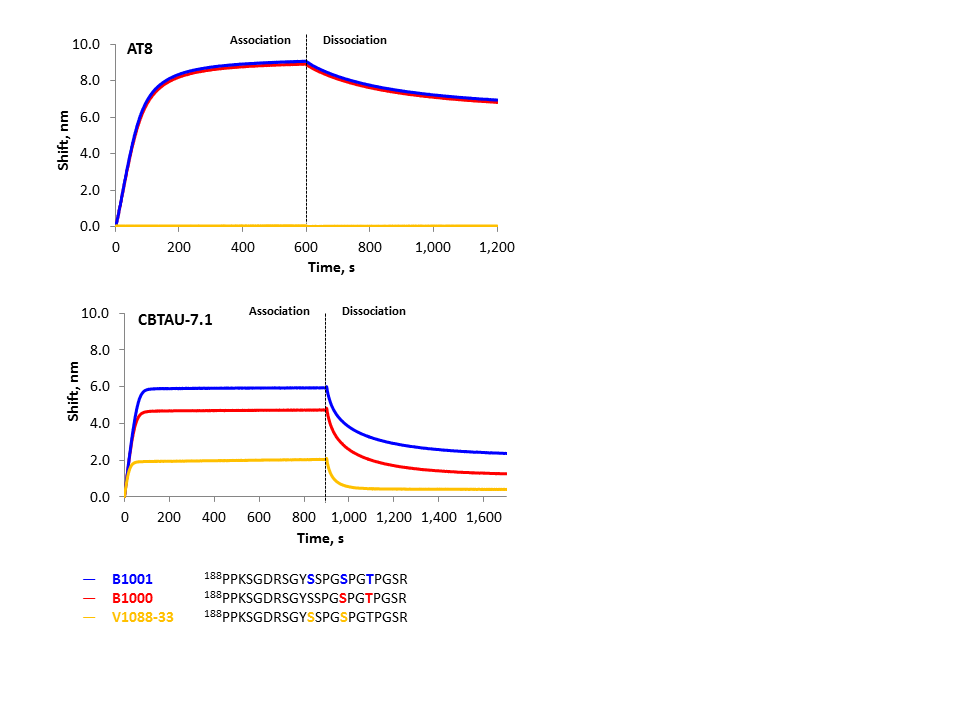

Supplement: Supplementary file 9 — Supplementary material 9 (TIFF 88 kb) [file 401_2017_1705_MOESM9_ESM.tif]

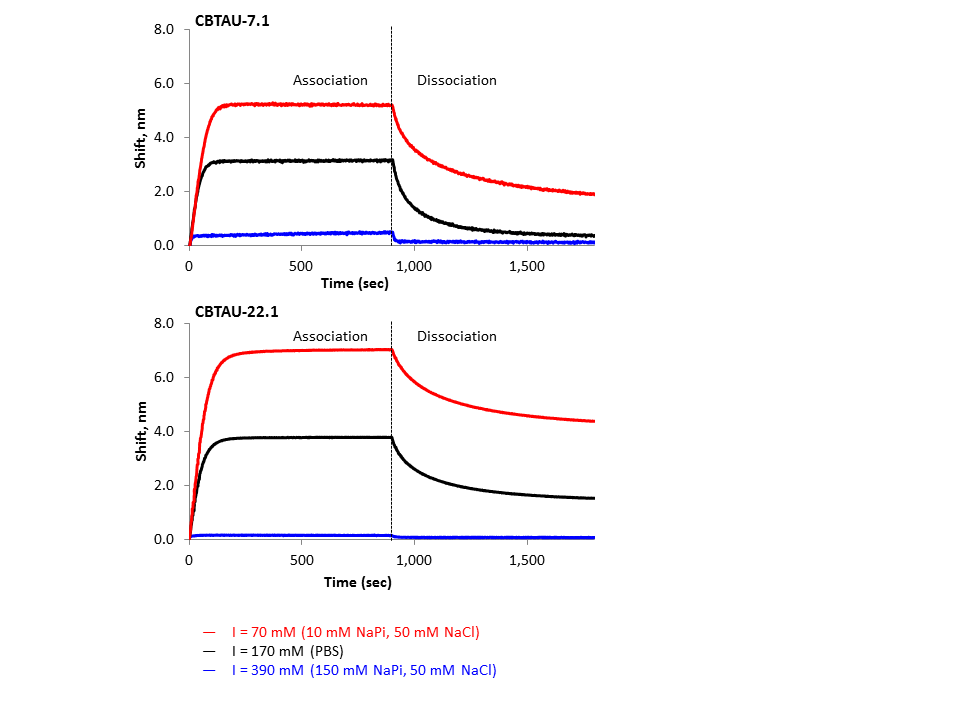

Supplement: Supplementary file 10 — Supplementary material 10 (TIFF 90 kb) [file 401_2017_1705_MOESM10_ESM.tif]

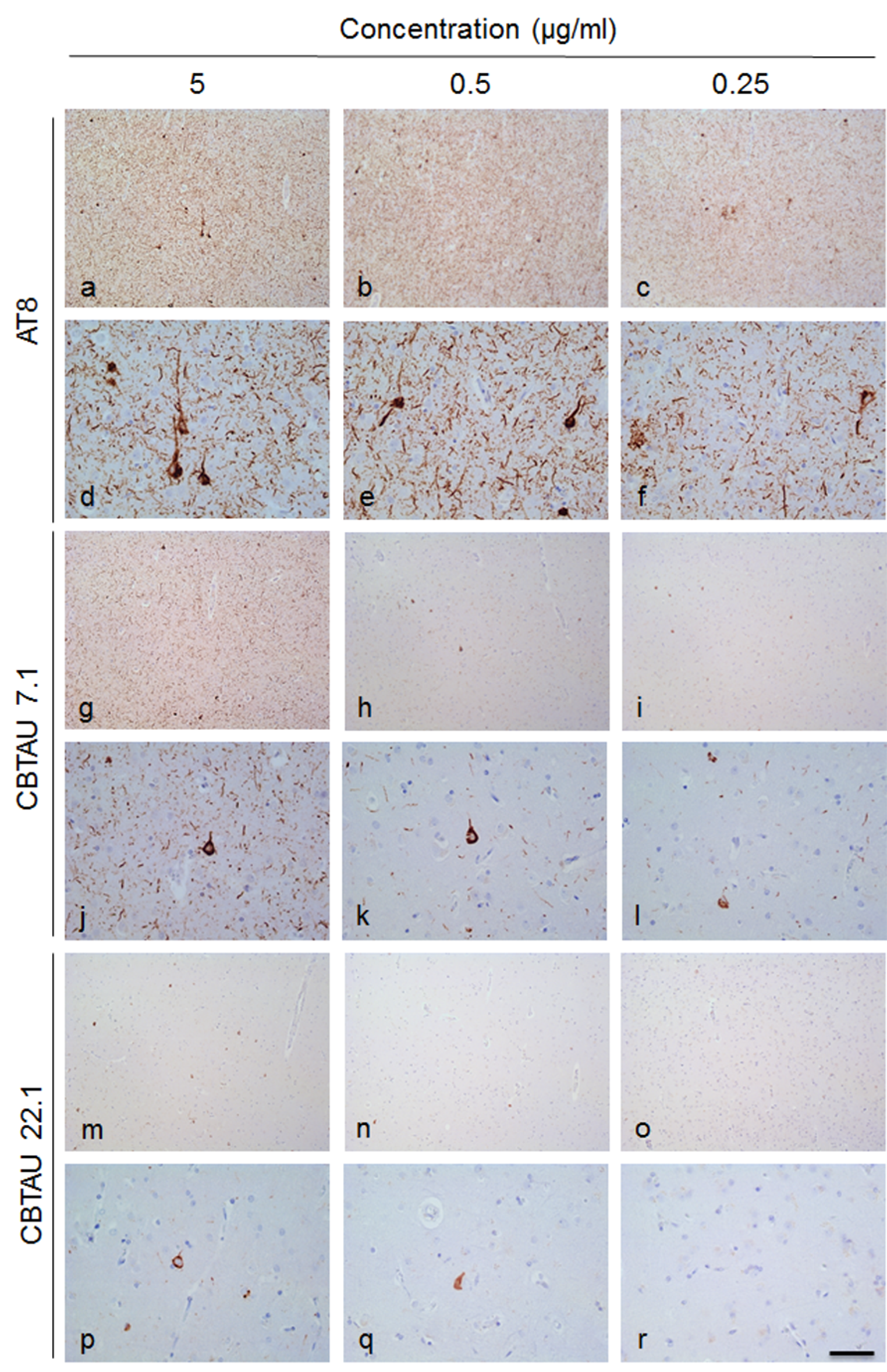

Supplement: Supplementary file 11 — Supplementary material 11 (TIFF 18857 kb) [file 401_2017_1705_MOESM11_ESM.tif]

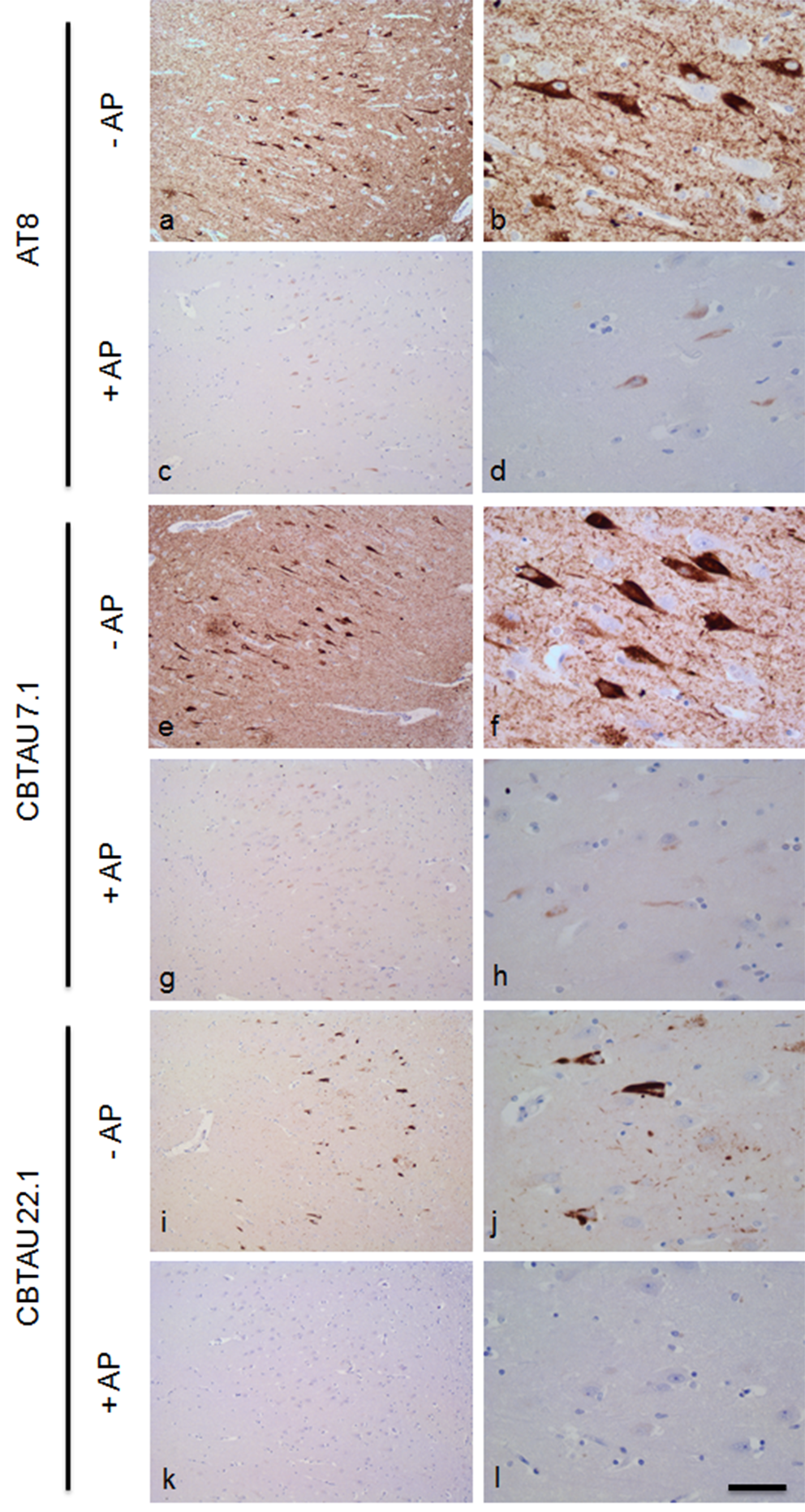

Supplement: Supplementary file 12 — Supplementary material 12 (TIFF 9863 kb) [file 401_2017_1705_MOESM12_ESM.tif]

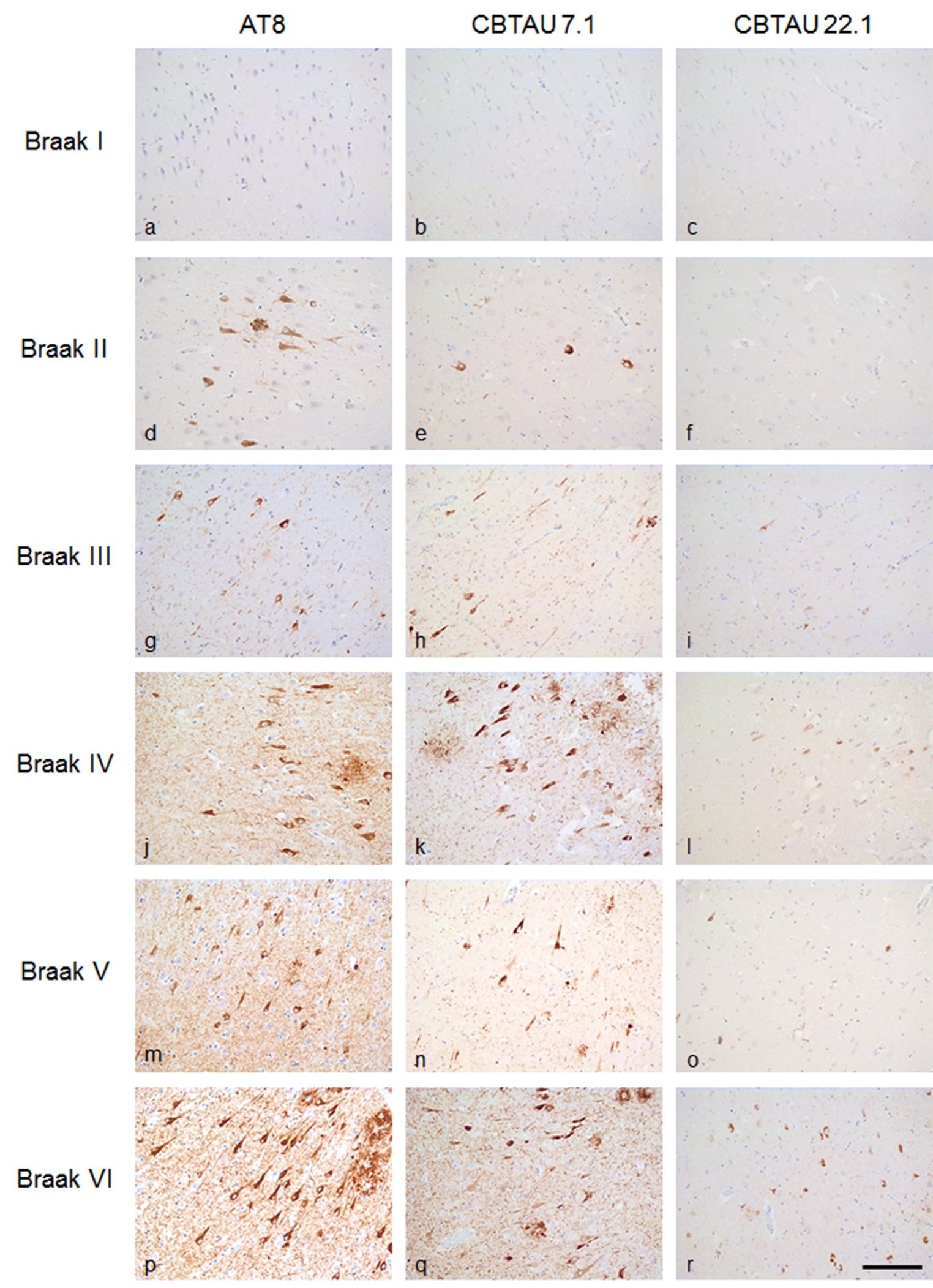

Supplement: Supplementary file 13 — Supplementary material 13 (TIFF 16385 kb) [file 401_2017_1705_MOESM13_ESM.tif]

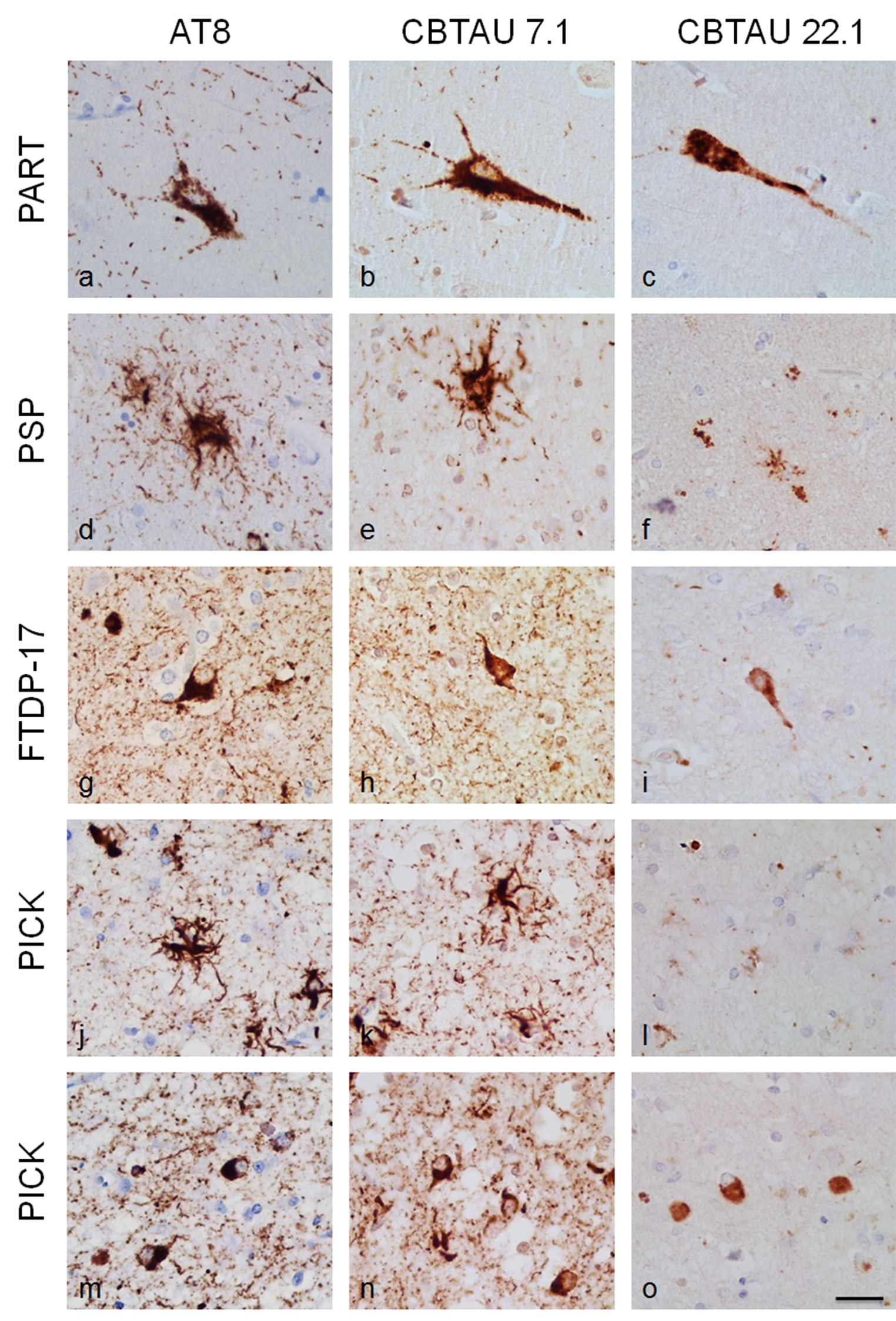

Supplement: Supplementary file 14 — Supplementary material 14 (TIFF 18488 kb) [file 401_2017_1705_MOESM14_ESM.tif]
